# Extensive T-Cell Epitope Repertoire Sharing among Human Proteome, Gastrointestinal Microbiome, and Pathogenic Bacteria: Implications for the Definition of Self

**DOI:** 10.3389/fimmu.2015.00538

**Published:** 2015-10-22

**Authors:** Robert D. Bremel, E. Jane Homan

**Affiliations:** ^1^EigenBio LLC, Madison, WI, USA

**Keywords:** T-cell repertoire, human proteome, microbiome, T-cell epitope, immunogenetics, central tolerance, *Mycobacterium*, *Burkholderia*

## Abstract

T-cell receptor binding to MHC-bound peptides plays a key role in discrimination between self and non-self. Only a subset, typically a pentamer, of amino acids in a MHC-bound peptide form the motif exposed to the T-cell receptor. We categorize and compare the T-cell exposed amino acid motif repertoire of the total proteomes of two groups of bacteria, comprising pathogens and gastrointestinal microbiome organisms, with the human proteome and immunoglobulins. Given the maximum 20^5^, or 3.2 million of such motifs that bind T-cell receptors, there is considerable overlap in motif usage. We show that the human proteome, exclusive of immunoglobulins, only comprises three quarters of the possible motifs, of which 65.3% are also present in both composite bacterial proteomes. Very few motifs are unique to the human proteome. Immunoglobulin variable regions carry a broad diversity of T-cell exposed motifs (TCEMs) that provides a stratified random sample of the motifs found in pathogens, microbiome, and the human proteome. Individual bacterial genera and species vary in the content of immunoglobulin and human proteome matched motifs that they carry. *Mycobacteria* and *Burkholderia spp* carry a particularly high content of such matched motifs. Some bacteria retain a unique motif signature and motif sharing pattern with the human proteome. The implication is that distinguishing self from non-self does not depend on individual TCEMs, but on a complex and dynamic overlay of signals wherein the same TCEM may play different roles in different organisms, and the frequency with which a particular TCEM appears influences its effect. The patterns observed provide clues to bacterial immune evasion and to strategies for intervention, including vaccine design. The breadth and distinct frequency patterns of the immunoglobulin-derived peptides suggest a role of immunoglobulins in maintaining a broadly responsive T-cell repertoire.

## Introduction

A central theme of immunology is the ability of the adaptive immune system to differentiate self from non-self. Much of the ability to mount a specific immune response is dependent on T-cell recognition of cognate peptide epitopes in the context of the MHC molecules determined by the host’s genetics. In this analysis, we examine the limitations of the T-cell epitope repertoire in distinguishing self from non-self by categorizing and comparing the TCEM repertoire of two groups of bacterial proteomes with that of the human proteome and immunoglobulins.

**Figure 1:**
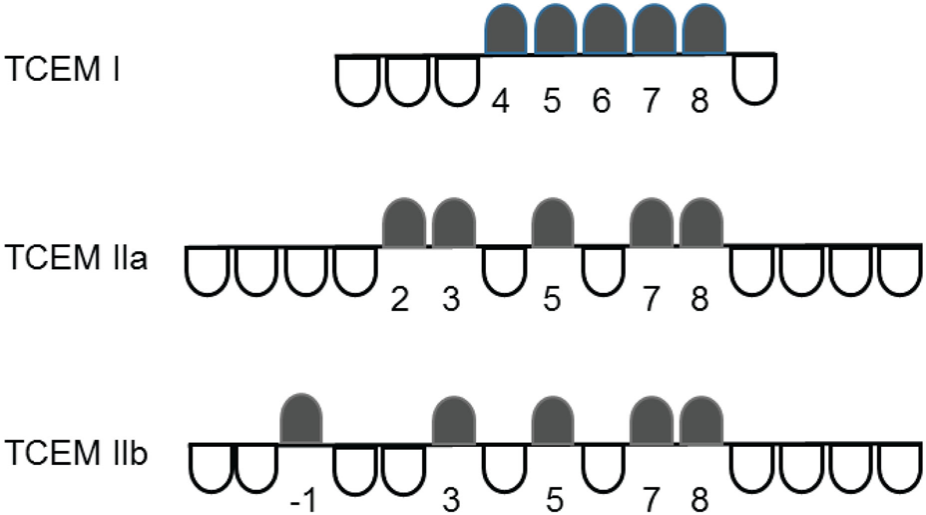
**T-cell-exposed motif patterns**. In each contact pattern the black markers indicate the T-cell exposed amino acids, while the white markers indicate the groove exposed amino acids. The numbers identify amino acids positions in each motif using the MHC groove pocket numbering scheme.

We have previously reported on the characteristics of T-cell exposed amino acid motifs of peptides bound in MHC molecules in mediating T-cell recognition and polyspecificity (1). T-cell exposed motifs (TCEMs) are the combinations of amino acids, typically pentamers, whose side chains are exposed to T-cell receptors when an epitope peptide is bound within a MHC groove. A TCEM is exposed to the T-cell within the context of the surrounding MHC histotope. Conversely, some of the amino acid side chains of the MHC-bound peptide are hidden to the T-cell receptor and are directed into the MHC groove, where they determine the binding affinity of the peptide to the MHC and thus the kinetic stability, or dwell time, during which T-cell receptor engagement occurs. Based on the structural analysis of Rudolph et al., three pentameric contact patterns of TCEMs are recognized (2). For MHC I, these comprise amino acids 4,5,6,7,8 of a core 9-mer, and for MHC II two patterns, which we call TCEM IIa and IIb, comprising the amino acids 2,3,5,7,8 and -1,3,5,7,8 of the central 9-mer core of a 15-mer (1, 2). These contact patterns are summarized in Figure 1. A pentamer contact pattern sets the maximum number of TCEM configurations that T-cells recognize at 20^5^, or 3.2 million, for each of the contact patterns. This determines the need for polyspecificity, and enables a finite T-cell population to respond to all potential epitopes (3, 4).

T-cell exposed motifs provide a framework to understand large protein sets from the perspective of T-cell recognition. We showed previously that in IgV the amino acids making up the TCEM assort independently of the amino acids making up the intercalated groove exposed motif (GEM) (1). The T-cell engagement is with the histotope and the exposed amino acids of the MHC-bound peptide, but not with the groove exposed amino acids. The T-cell–TCEM interaction is not influenced by the GEM except in so far as the latter determines the binding affinity and thus how long the peptide remains associated with the MHC to enable repeated T-cell interactions to occur. Therefore, we approached this analysis by looking first at the TCEM motifs alone.

For any protein, there is a gradation in frequency of T-cell encounter with different TCEMs present in that protein. Common TCEMs will cause repeated clonal stimulation leading to a buildup of a larger population of cognate T-cells, whereas rare TCEM will have fewer encounters and generate a smaller cognate population (4). The motif usage patterns previously observed in the IgV prompted us to examine the patterns of occurrence of TCEMs in other large protein sets relevant to the function of the immune system: the human proteome exclusive of the immunoglobulins, and two exemplary groups of bacteria. The first group of bacteria comprises 67 species in 35 genera from the gastrointestinal microbiome. The second group comprises up to five proteomes each of 28 species in 12 genera of pathogenic bacteria. We characterized the patterns of potential TCEM present within each proteome dataset, and compared them to each other, and to the repertoire of motifs in IgV regions.

The interdependency of the B-cell immunoglobulin variable regions (IgV) and T-cell suppressor repertoires has long been recognized (5). Peptides derived from IgV are processed, bound in MHC, and presented to T-cells as are other peptides of other origins (6–8). B-cells present their own endogenous IgV-derived peptides in MHC (9), a process that is thought to play a key role in determining T-cell diversity (10, 11). We reported that within IgV regions there is a high rate of repetitive appearance of TCEM of both germline-origin and those resulting from somatic hypermutation (1). In a sample of “normal” IgV, from which sequences originating from immunopathologies had been eliminated, estimated to be approximately equivalent to 1% of the population of B-cell clonotypes in any one individual (12), we showed that approximately 50% of the TCEMs recur in at least 1 in every 1024 B-cell clonal products. We proposed that the peptides derived from IgV, and presented on MHC by B-cells and by other antigen presenting cells (APCs), provide the constantly cycling peptide diversity needed to maintain an active T-cell repertoire required to mount an appropriate immune response to a vast diversity of antigens, both self and non-self, of pathogen or environmental origin.

We find that the human proteome uses only about three quarters of the total possible pentamers in each TCEM contact pattern and thus the immune system has evolved mechanisms to recognize and respond appropriately to the other quarter of the motifs. The bacterial species share motifs with the human proteome but in addition contain nearly all of the TCEMs not found in the human proteome, a characteristic that highlights the importance in immune recognition of the sampling of the microbiome by the gut associated lymphatic tissue. The IgV-derived TCEMs provide a broad representation of the other proteomes and also generate TCEMs not found in any of the three proteome datasets. Within the bacterial pathogen dataset we can differentiate protein-specific, genus-specific, and some species specific patterns in TCEM usage. Overall, the relationship points to the role of TCEM frequency in pathogen immune evasion, tolerance of commensals, and the potential for generation of autoimmune responses following bacterial infection or disturbance of the microbiome.

## Materials and Methods

### Protein Sets

Four groups of protein sequences were assembled:

- Human proteome (human proteome): this group comprises 88,146 proteins, including multiple isoforms of proteins, with an average length of 384 amino acids. As a whole, the set thus provides about a fourfold redundancy of the approximately 20,000 proteins in any individual human proteome. This set of proteins was assembled from the UniProt repository^1^ (13), and based on the UniProt annotations it was manually curated to remove the proteins containing immunoglobulin variable regions. The final curated set comprised 3.3 × 10^7^ total TCEM in each of the three contact patterns.
- Gastrointestinal microbiome bacteria (microbiome): the proteomes were assembled from the NIH Human Microbiome Project Reference Genomes database^2^ and included 67 species in 35 genera (listed in Table S1 in Supplementary Material). This set comprised a total of 378,061 proteins with an average length of 290 amino acids. This is not a set of fully annotated proteomes and may include some partial proteomes. The final set comprised 1.09 × 10^8^ total TCEM in each of the three contact patterns and is thus of a comparable total size to the bacterial pathogen set in (c).
- Exemplary bacterial pathogens (pathogens) comprising 28 species each represented by up to five proteomes (132 in all) selected at random from the complete proteomes of each genus available at PATRIC^3^ (14). The genera included were: *Bordetella, Brucella, Burkholderia, Chlamydia, Clostridium, Coxiella, Francisella, Mycobacterium, Neisseria, Staphylococcus, Streptococcus*, and *Ureaplasma*. Species and strains are shown in Table S1 in Supplementary material. Proteomes ranged in size from approximately 6000 proteins (*Burkholderia*) to 689 (*Ureaplasma*). In total 427,906 proteins were included in the final pathogen dataset, comprising 1.16 × 10^8^ total TCEM in each of the three TCEM contact patterns.
- Immunoglobulin variable regions (IgV): the 40,000 heavy chain V regions were assembled as previously described (1). A further 16,000 light chain variable regions were also retrieved from Genbank and curated to remove those derived from immunopathologies, using the same criteria as described for the heavy chains. The final reference databases comprised approximately 6.4 × 10^6^ total TCEM, including 325,000 unique motifs in each of the three contact patterns.

### Analysis of T-Cell Exposed Motifs

To construct the TCEM sets, concatenated amino acid FASTA files of each of the proteomes were assembled, from which each protein in the proteome was then decomposed into sets of 9-mer and 15-mer peptides, each offset by a single amino acid and placed in successive rows of a JMP^®^ data table. Three different TCEM contact patterns were constructed from each peptide in corresponding columns using column formulas of JMP^®^. These were TCEM I comprising amino acids (4,5,6,7,8) of a core MHC-I 9-mer, and TCEM IIa and TCEM IIb comprising amino acids corresponding to amino acids (2,3,5,7,8) and (-1,3,5,7,8) of the central 9-mer core of a 15-mer (1, 2). Hence, each 9-mer yielded a motif in the format ~~~~XXXXX~ where ~ represents a non-exposed amino acid, and each 15-mer yielded a motif in the format ~~~~XX~X~XX~~~~ or ~~X~~X~X~XX~~~~. The GEM responsible for the binding interactions was similarly assembled as the intercalated set of non-exposed amino acids in each peptide. The unique identifier of each protein, the peptide, and the N terminal positions of each peptide in the parent protein within the proteome were retained with each TCEM. In this way TCEM sets were hyperlinked to the parental proteins by JMP^®^ so that the parental proteins could be readily retrieved for other analyses. Three sets of tables, one for each TCEM contact pattern, were constructed for each protein set.

### Frequency and Diversity Analysis of Proteome TCEM

Preliminary analysis of TCEM frequencies in the different proteomes indicated that they all exhibited a log-normal distribution pattern characterized by two parameters: a mean (μ) and a scale factor (σ) analogous to an SD. In keeping with our previous use of a log2 base for characterizing the frequency-of-occurrence of TCEM in IgV (1), each motif was characterized according to the frequency of occurrence in each particular proteome. The diversity of TCEM occurrence in each proteome was computed as the Shannon Entropy H as

$$H=-\sum_{1}^{N}p\;\log2\;p$$

where *p* = frequency of occurrence of a particular TCEM and *N* = total number of TCEM in the proteome for the contact pattern.

### TreeMapping of Multivariate Datasets

Treemaps were used to visualize underlying patterns in large multivariate datasets and were particularly useful for displaying hierarchical data as a set of nested rectangles. Briefly, TCEM usage in each proteome was tallied for each of the proteomes. Each of the proteomes exhibited a pattern of motif use, some of which was shared with other organisms and the human proteome, and some of which was unique to the particular organism. An *N* × *M* matrix where *N* = 3.2 million and *M* = the number of proteins was created which contained the tallies of the number of each of the particular TCEM. Then a binary string was created which described the presence or absence of each of the motifs across all proteomes. This string pattern was used to create a treemap of comparative TCEM content across proteomes. This process works well for a number of proteomes within a genus, and across genera and provides a useful way to compare and visualize large proteomic datasets.

### Immunoglobulin TCEM Frequencies

The TCEM in each of the proteomes described were characterized as to whether or not they were present in immunoglobulins and, if present, their particular frequency of occurrence in immunoglobulins. It should be noted that in this paper we use the frequency of TCEM occurrence based on total TCEM within the whole immunoglobulin dataset. This differs from our prior analysis where only IgV data was analyzed (1), and hence where frequencies were standardized to the number of IgV clonotypes.

### Software

All data processing, pattern analysis and statistical analysis were done with JMP^®^ v12 from SAS Institute (Cary, NC, USA).

## Results

All data described in the Section “Results” are for TCEM IIa, corresponding to MHC II mediated CD4+ responses. The corresponding results of analysis for TCEM I, for MHC I CD8+ responses, is appended in the Supplemental Tables and Figures. Results for TCEM IIb are not shown but are very closely analogous to TCEM IIa, as are results observed for all three contact patterns.

### TCEM Content and Frequency Distribution in Human Proteome, Microbiome, and Pathogen Datasets

**Figure 2:**
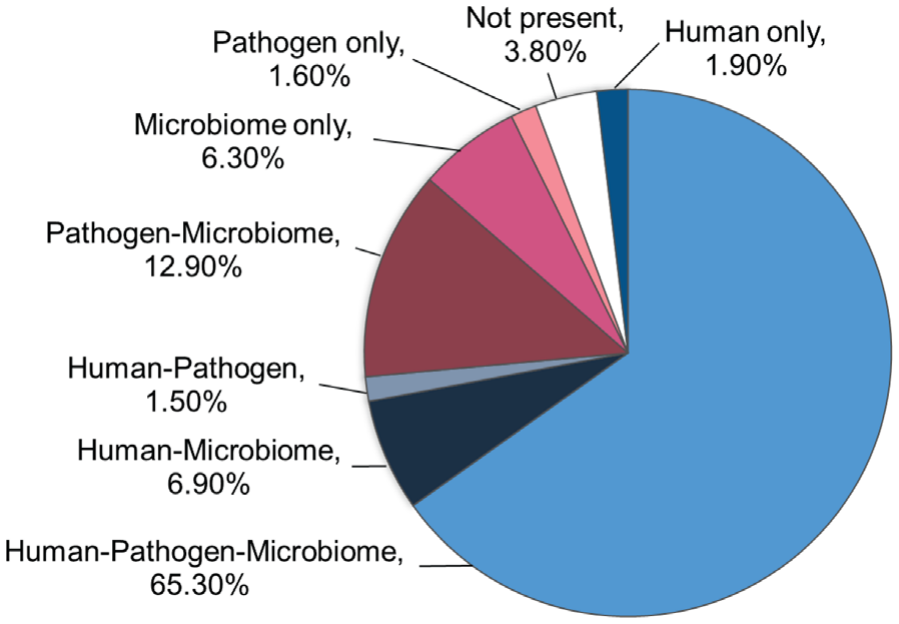
**Occurrence of the 3.2 million possible motifs**. The chart shows the distribution of the maximum possible TCEM IIa pentamer motifs among the three proteome datasets.

Based on the pentamer contact pattern, there is a maximum of 20^5^ possible TCEMs (3.2 million) for each of the three contact patterns TCEM I, IIa and IIb. Table 1 shows the frequency distribution of TCEM IIa in each proteomic dataset. None of the proteome datasets uses the full complement of possible TCEMs and there is a varying degree of overlap between proteome datasets. The Human proteome dataset comprises 2,412,699 unique TCEM, or only about three quarters of the maximum possible motifs (Table 2; Figure 2). Of these, 2,090,606 are shared with both the Microbiome and Pathogen composite proteome sets. The Human proteome shares a further 216,262 motifs with only the Microbiome. A smaller set of 46,575 additional TCEMs is shared by only the Human proteome and the Pathogens. The Pathogen set and the Microbiome sets share 413,177 motifs that are missing from the Human proteome, however this reflects a more complex underlying species-specific relationship (Tables S6 and S7 in Supplementary material). Only 59,256 motifs are completely unique to the Human proteome and not found in the bacterial sets. A final 121,683 possible motifs are not used by any of the proteome sets, as discussed further below.

**Table 1:** **Composition of TCEM IIa proteome datasets**.

TCEM count	Human	Pathogen	Microbiome	Percent of 3.2 million
2,090,606	1	1	1	65.3
216,262	1	0	1	6.9
413,177	0	1	1	12.9
201,400	0	0	1	6.3
46,575	1	1	0	1.5
59,256	1	0	0	1.9
51,041	0	1	0	1.6
121,683	0	0	0	3.8

**Table 2:** **Fractional composition of proteomic datasets and fractional matches of IgV TCEM IIa motifs**.

Group	Count	Percent of 3.2 million	IgV coverage	IgV motifs	Percent of total IgV motifs
Human	2,412,699	75.4	11.6	279,108	85.9
Human excluded^b^	787,301	24.6	5.5	42,950	13.3
Pathogen	2,601,399	81.3	11.5	298,764	92.8
Pathogen excluded^b^	598,601	18.7	3.9	23,294	7.2
Microbiome	2,921,445	91.3	10.7	313,197	96.4
Microbiome excluded^b^	278,555	8.7	3.2	8,861	2.7
Not found in human, pathogen or microbiome^a^	121,683	3.8	2.4	2,900	0.9

*^a^This set is included in the three excluded sets marked “b”*.

*^b^Excluded sets*.

**Figure 3:**
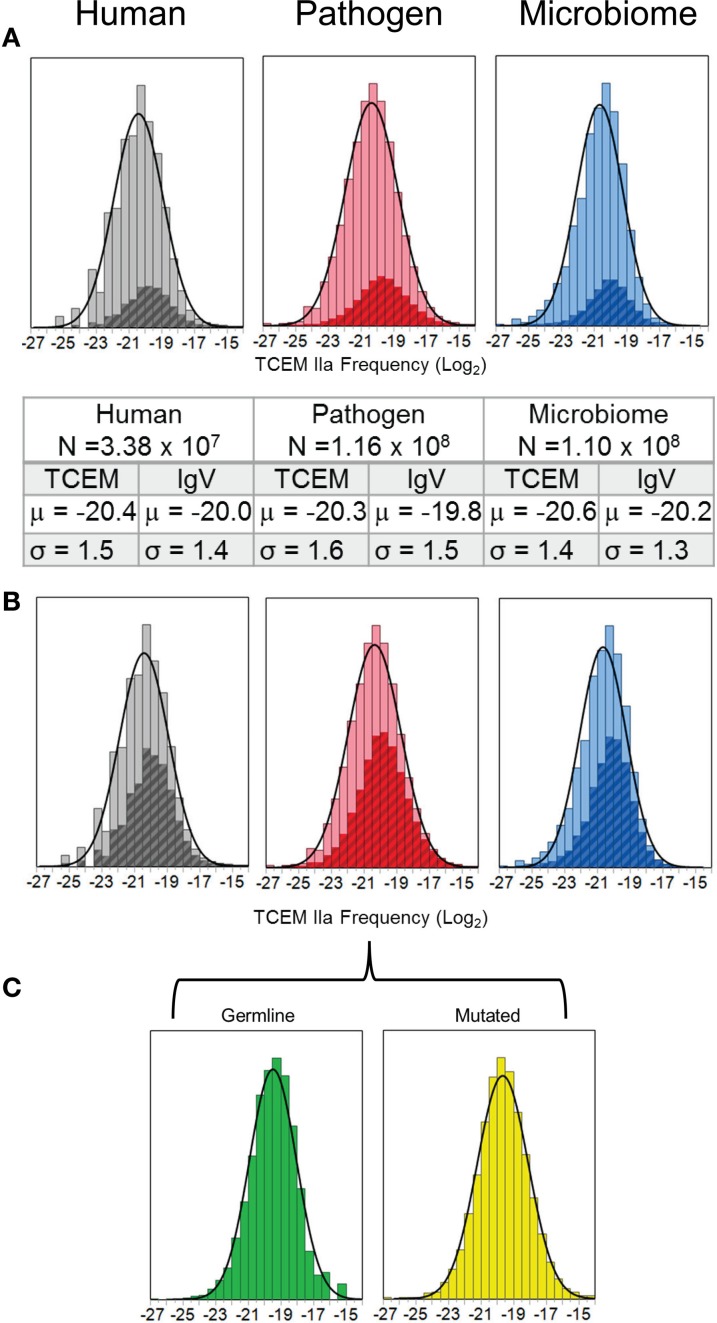
**Histograms of TCEM IIa in each proteome set analyzed**. **(A)** The frequency of occurrence of each of the possible 20^5^ TCEM IIa pentamers was computed for all of the proteins in each of the proteomes. The fit of a frequency-weighted normal distribution to each of the datasets is shown by the solid curve. The number of motifs and the parameters of the normal distribution curve are given in the inset. The cross hatched region within the histogram corresponds to the frequency of IgV-origin TCEM IIa. *X* axis shows the log2 of frequency of occurrence. **(B)** The background frequency distribution is as in **(A)** but showing the overlap of IgV TCEM IIa pentamers when a single conservative amino acid replacement is permitted. **(C)** Separation of the IgV origin pentamers for the Pathogen proteomes to show germline-origin and somatic hypermutated-origin subset distributions of the IgV TCEM IIa.

The frequency distribution of motif usage within each of the three proteome sets is log-normal (KSL goodness of fit *p* < 0.001 (15). As is shown in Figure 3A both the mean (μ) frequency and the variation (σ) in occurrence are similar between the three different proteomic data sets. The log2 mean of the distributions is about μ = −20 (μ = −20.3 for human proteome) for each of the proteomes (1/2^20^ = 1/1,048,576) or a somewhat greater frequency than 1/3,200,000 that would be expected for a totally random distribution. Moreover, a very slight skewing of the histograms can be seen relative to the overlayed normal distribution curve. The crosshatched insets are the IgV TCEM frequency distribution, which also show a log-normal distribution. The size of the IgV database used in this analysis is approximately 1% of the total clonotypes that might be found in a single human (12), but even this relatively small sample of total clonotypes provides a considerable intersection with the proteomic data (Table 2). Additionally, allowing for a single conservative amino acid replacement (16, 17) at any of the five amino acid positions in the TCEM pentamer expands coverage by the IgV-derived motifs to about half of the total motifs in the proteomes (Figure 3B). Comparison of the distribution means in Figures 3A,C shows that in each case the mean of the IgV distribution is slightly shifted toward a higher TCEM frequency as compared to the mean of the proteome as a whole (μ difference 0.4–0.5 log2).

### Motif Usage in Immunoglobulin Variable Regions

**Figure 4:**
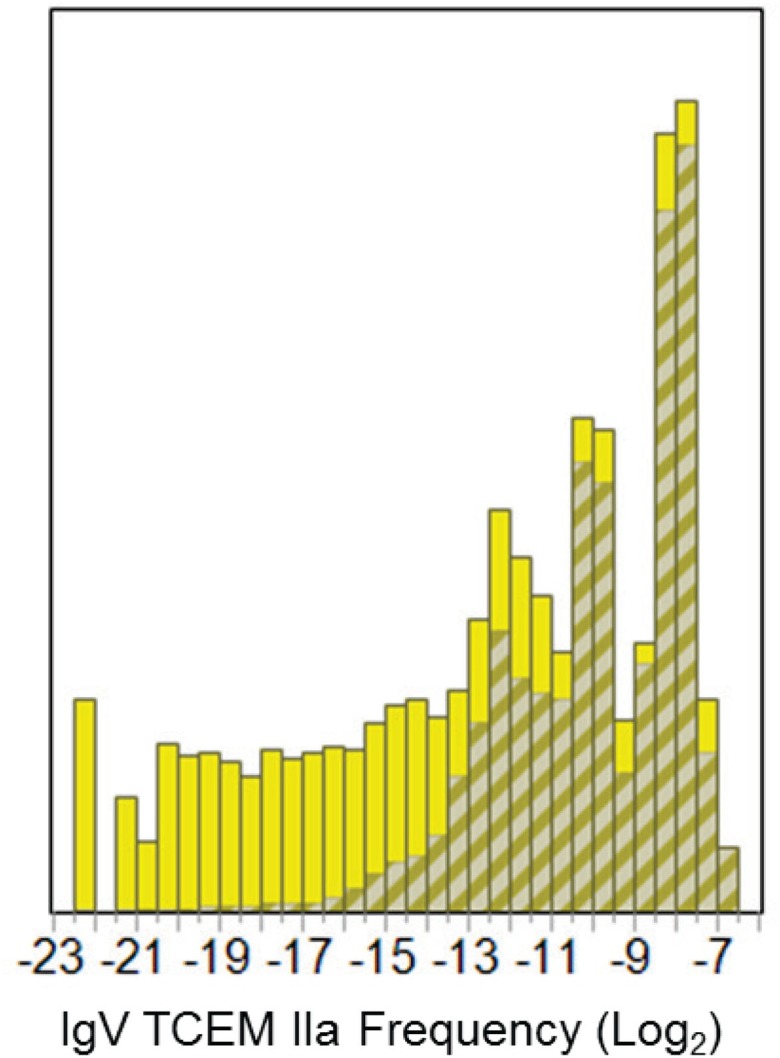
**Frequency distributions of IgV-origin TCEM IIa motifs for the Pathogen proteome**. Shows the frequency distribution of the IgV TCEM IIa motifs in the Pathogen proteome set as shown by the cross hatched insets in Figure 3A. The bars in the histogram represent the fraction of the total motifs at the particular frequency. The crosshatched region depicts the fraction of the total at the particular frequency that is of germline-origin and the solid portion of the bar is attributable to somatic hypermutation origin motifs. *X* axis shows the log2 of frequency of occurrence.

The log-normal pattern of TCEM recurrence frequency seen in the three proteome data sets, described above, is very different from the frequency of occurrence pattern of motifs found in the IgV, and as previously reported (1). The IgV dataset comprises only 322,058 unique motifs in the TCEM IIa contact pattern but these have a distinctive frequency distribution pattern of motif re-use. The overall frequency of germline-origin and somatic hypermutation-origin sequences is seen in Figure 4.

The different underlying basis of the diversity in the IgV, the Human proteome and the two bacterial proteomes is evident in the Shannon entropy of the TCEM motifs in the different datasets (Table 3). Since the frequency of occurrence of TCEM is the base of the metric, the entropy calculation is standardized for dataset size, so the values derive from the differences between the combinatorial frequency patterns of the IgV and the proteomes. The difference in patterns as well as the differences in entropy can be understood by the fact that the IgV begins as the same germline set (albeit from a number of different gene families) which becomes increasingly diverse during the course of somatic hypermutation and thus comprises highly diverse sequences but which are systematically created from a constant starting point.

**Table 3:** **Shannon entropy of TCEM IIa motifs in different proteome subsets**.

Source	Shannon entropy (*H*)
Human and pathogen	20.3
Human and microbiome	20.6
Human	20.4
IgVL	12.3
IgVH	12.5

Given the differences in overlap between proteome sets, we were interested to examine what the degree of overlap with TCEM used in IgV was for each proteome. At a full dataset scale there was a slight bias in the IgV matches toward the more commonly encountered motifs (Figure 3A). We compared the frequency of usage of TCEMs in each of the three proteome data sets to that in the IgV dataset by subsetting the proteomes into the discreet IgV log2 based frequency categories (FC) as previously described (1). In this system TCEM motifs are characterized by the number of B-cell clonotypes that display the particular motif; hence a motif designated as FC3 occurs in 1 in 8 B-cell clonotypes and a motif of FC10 occurs in 1 in 1024.

We found that every FC subset of IgV TCEM matched a random sub-sample of each of the three of the proteomic datasets (Figure S3 in Supplementary Material). There is a slight tendency toward the high frequency FC motifs from IgV having greater overlap with the higher frequency motifs in the Human proteome (μ = −18.2 for FC7 vs. μ = −19.7 for FC12). Overall, however, there is a substantial overlap with the full gamut of different frequency peptides in the three proteome sets. The IgV frequencies thus effectively comprise a stratified random sample of TCEMs that the immune system will encounter. Motifs from the three proteomes are randomly distributed within each of the IgV FC categories.

That there is a slight bias in the coverage of IgV can be seen in a different way in Table 2; the IgV TCEM database has a total of 279,108 matches of the 2,412,699 motifs in the Human proteome, an 11.6% match. This match is with 85% of the IgV database, which in total approximates only 1% of total B-cell clonotypes that an individual might carry. This suggests that with a complete B-cell repertoire, there might actually be a redundant level of motif matching across all Human Proteome motifs. The bias of IgV matches toward the more commonly used motifs is likewise seen in the Pathogens (11.5%) and in the Microbiome (10.7%). Thus in each proteome, while the IgV match seems to favor the more commonly encountered motifs, which are present more often than 1 in 1024 IgV clonotypes, the somatic hypermutation process is evidently capable of generating a range of motifs beyond what are found in the TCEM of the Human and bacterial proteomes.

We hypothesized that commonly used pentamers might serve some consistent structural or functional role in proteins. If so, then the relative frequency of occurrence of particular pentamer motifs would be the same regardless of the source organism as motifs that serve critical physicochemical roles in protein structure would be expected to occur in either bacteria or man at similar frequencies. To evaluate this we computed the overall rank correlation of usage frequency in the different proteomes (Table 4). The Spearman rank correlation in motif usage between Microbiome and Pathogen proteomes is 0.7. Although quite high, it is clearly substantially less than unity. The rank correlation was lower still between either of the bacterial sets and the Human proteome at approximately 0.5 indicating that human and bacteria comprise different patterns of TCEM in building proteins. The rank correlation between IgHV and IgLV motif usages is 0.27 but the correlations of usage between the IgV and all of the proteomes is very low (0.03–0.06), indicating no structural or functional relationship of IgV to the proteome sets.

**Table 4:** **Rank correlation of TCEM IIa occurrence between different proteomic subsets**.

Variable	By variable	Spearman ρ	Prob > |ρ|
log2 Human freq	log2 Microbiome freq	0.5249	<0.0001
log2 Pathogen freq	log2 Microbiome freq	0.7404	<0.0001
log2 Pathogen freq	log2 Human freq	0.5060	<0.0001
log2 IgVL freq	log2 Microbiome freq	0.0621	<0.0001
log2 IgVL freq	log2 Human freq	0.0652	<0.0001
log2 IgVL freq	log2 Pathogen freq	0.0512	<0.0001
log2 IgVH freq	log2 Microbiome freq	0.0496	<0.0001
log2 IgVH freq	log2 Human freq	0.0382	<0.0001
log2 IgVH freq	log2 Pathogen freq	0.0372	<0.0001
log2 IgVH freq	log2 IGVL freq	0.2699	<0.0001

### Missing Motifs

**Figure 5:**
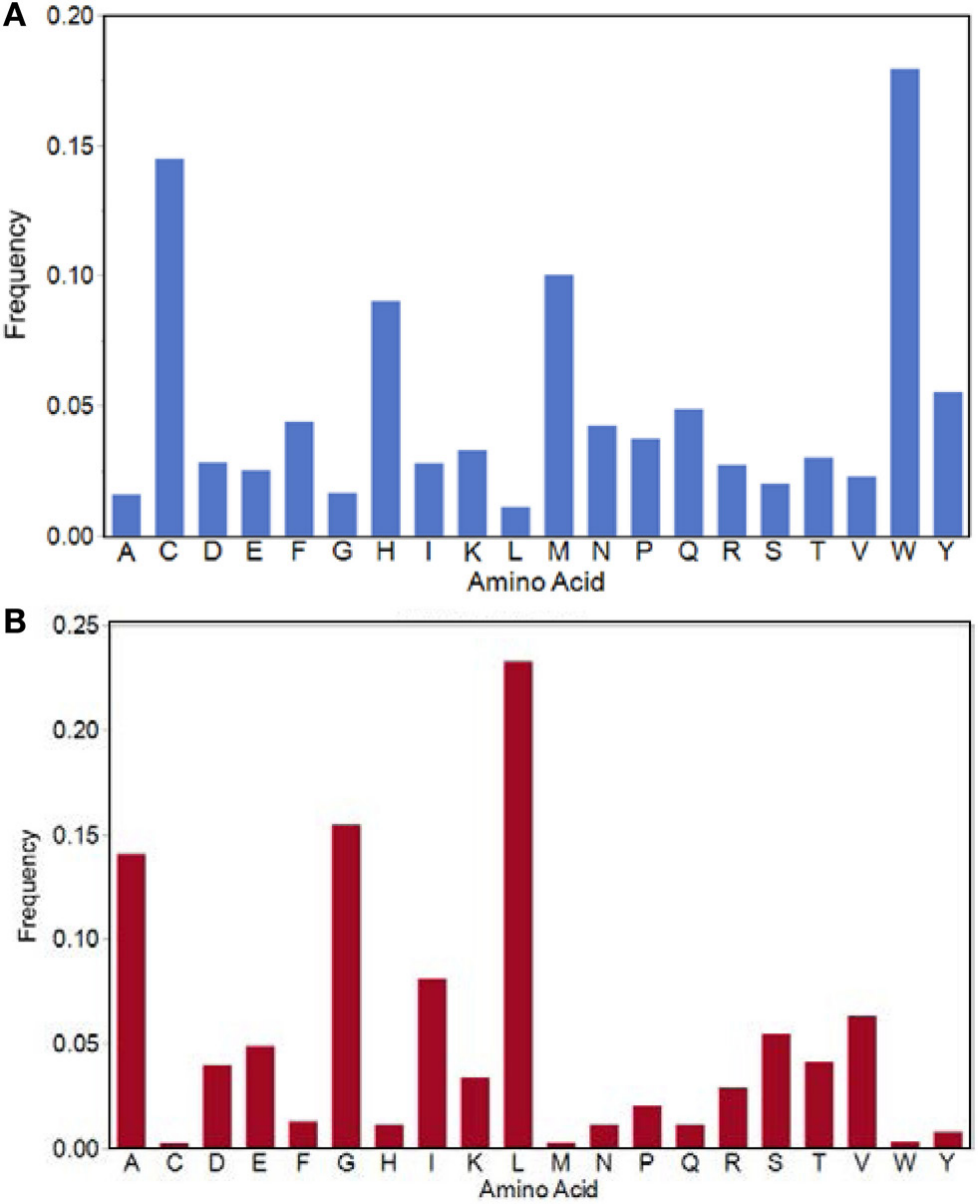
**Amino acid frequencies in the TCEM IIa pentamers**. **(A)** Amino acid frequency in the potential motifs which are not found in any of the proteomes evaluated. **(B)** Amino acid frequencies in the motifs used by all bacteria in both Pathogen and Microbiome sets and the Human proteome.

There are 121,683 motifs of the 3.2 million possible that are absent from all three proteome datasets (Table 1; Figure 2). The amino acid composition of this set of “forbidden” motifs comprises high percentages of tryptophan, methionine, cysteine, and histidine (Figure 5A). The overall coverage of these missing motifs by motifs found in the IgV dataset is 2.4% or 2900/121,683 and TCEM of all IgV FC are represented. Again, a complete B-cell repertoire would likely provide more complete coverage of even these rare motifs. Hence, the mutational process generating the IgV can create even this “forbidden” subset of TCEM that are not found in the Human proteome or in the very diverse bacterial proteome datasets.

### TCEM Frequency Patterns within the Bacterial Datasets

Although at a global level the individual bacterial proteomes making up the bacterial datasets are quite similar in TCEM occurrence frequency, there are clear differences in detail of the constituent bacterial proteomes that may be of immunological significance. We observed two independent types of patterns of TCEM occurrence in individual proteins in the bacterial sets:

(a) some genera comprise many proteins with a high percentage of IgV motif overlap, and
(b) each bacterial genus and some species exhibit a characteristic signature of motif usage and of motif overlap with the Human proteome.

#### Comparison of IgV TCEM Content in Pathogen and Microbiome Proteomes

**Figure 6:**
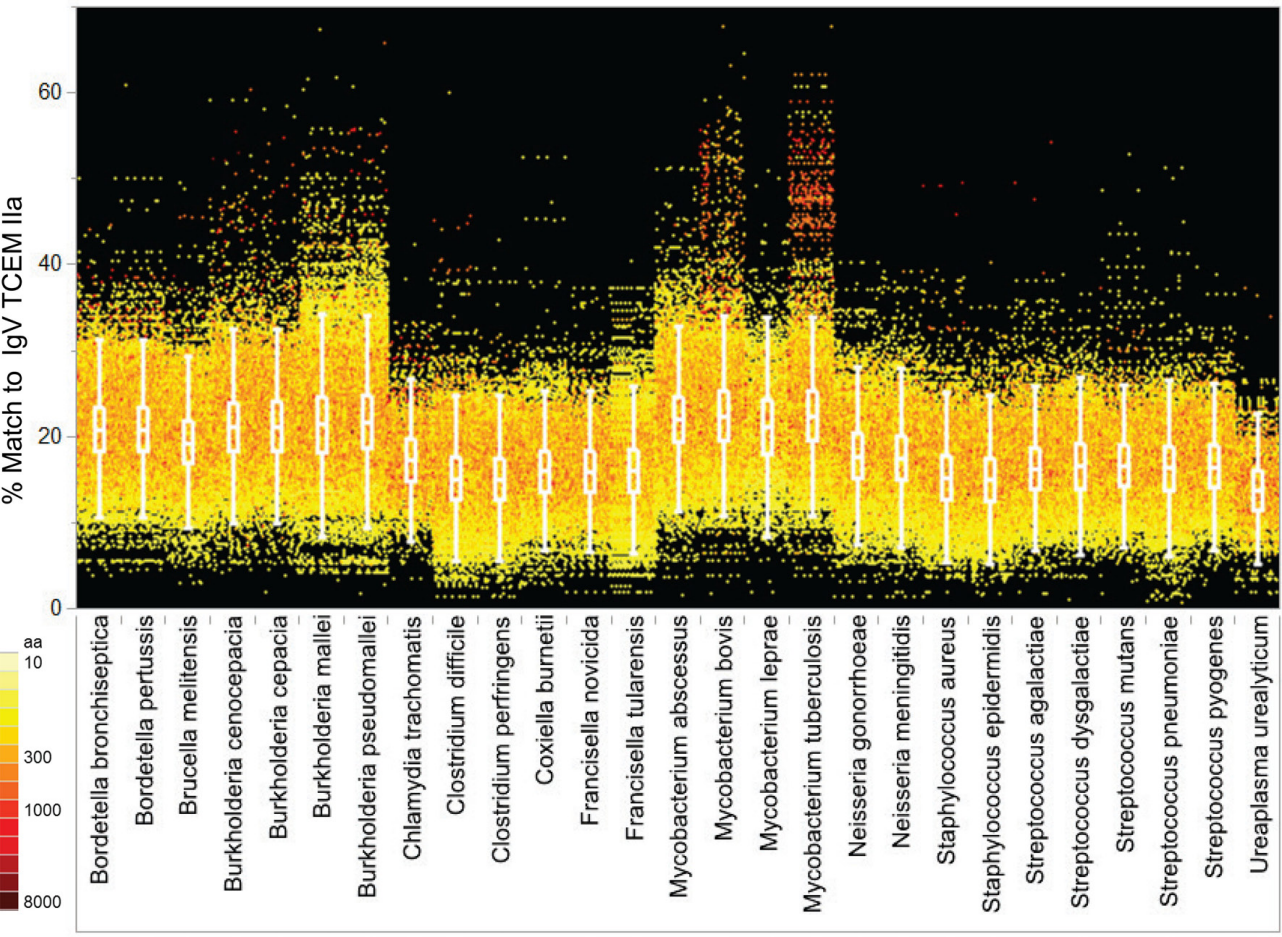
**IgV TCEM IIa frequency patterns within the Pathogen proteomes**. *X* axis: groups of five Pathogen total proteomes are clustered by genus with each protein represented by a colored dot. *Y* axis shows the percentage match of IgV TCEM IIa in each protein. Each dot represents the metric computed for a single protein in the proteome of the different organisms. The color of the dot is proportional to the molecular weight of the protein. For example, *Mycobacterium tuberculosis* has a number of relatively large proteins which have nearly a 50% match to the IgV motifs. The inset box shows the interquartile range of the metric in each of the proteomes and the whiskers indicate the 10 and 90% of the metric. For the human proteome the average content of IgV TCEM IIa is 17.3%.

As shown in Figure 6, when the proteomes of each bacterial Pathogen genus analyzed are arrayed by their IgV motif content, there are clear differences between genera and between species and the IgV motif found in the Human proteome (see also Table S6 in Supplementary Material for analysis of variance). In particular, *Burkholderia spp* and *Mycobacterium spp* exhibit a large number of proteins with up to 50% IgV motif content. As a group, the majority of the selected Pathogens have a higher content of TCEM shared with IgV than does the Human proteome. In *Burkholderia spp* and *Mycobacterium spp* in particular there are entire families of proteins that have high IgV similarity. The high IgV TCEM content is found in individual proteins and in classes of proteins sharing functional similarities. Proteins of some Pathogen genera have a lower overall IgV motif content, but each has a small number of proteins that are outliers with respect to their IgV motif content. As Figure 6 also indicates (see coloration) these outliers with higher IgV match include quite large proteins (>2000 amino acids) and IgV content is expressed as a percentage of TCEM, so their size might be expected to significantly contribute to their overall immunological effect.

**Figure 7:**
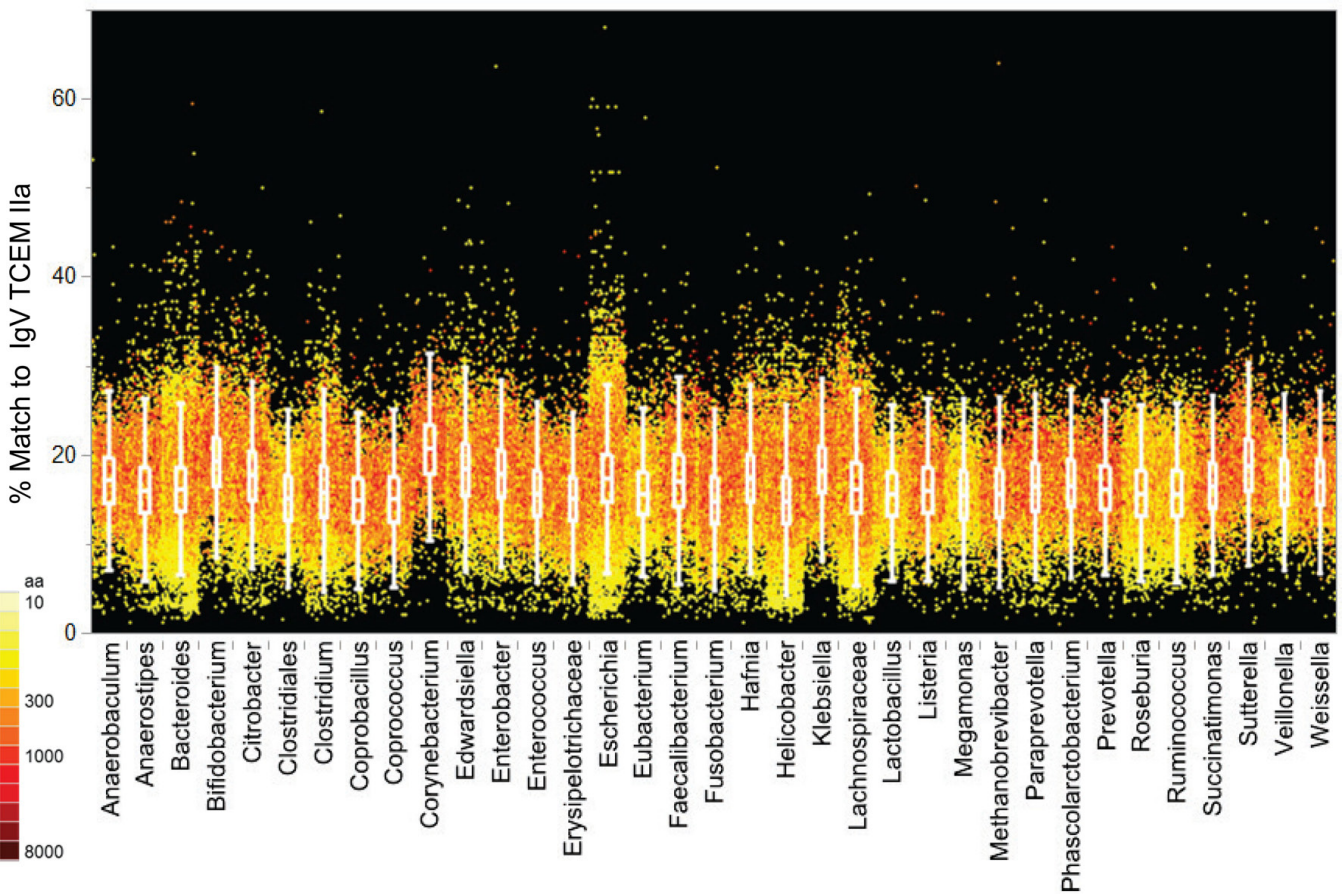
**IgV TCEM IIa frequency patterns in the Microbiome proteomes**. *X* axis: groups of Microbiome proteomes are clustered by genus with each protein represented by a colored dot. *Y* axis shows the percentage match of IgV TCEM IIa in each protein. Each dot represents the metric computed for a single protein in the proteome of the different organisms. The color of the dot is proportional to the molecular weight of the protein. The inset box shows the interquartile range of the metric in each of the proteomes and the whiskers indicate the 10 and 90% of the metric. For the human proteome the average content of IgV TCEM is 17.3%.

In contrast, applying a similar analysis to the Microbiome set (Figure 7; Table S7 in Supplementary Material) does not reveal differential distribution with respect to IgV motif content. All of the genera have similar patterns, although notably *Escherichia*, included in the Microbiome set, does have a slightly higher content of IgV motifs. In part the lack of distinct patterns among the Microbiome maybe attributable to the taxonomic diversity, however the IgV motif content is overall similar to the lower range seen in the Pathogen set.

To relate the IgV content to Human proteome content we applied the frequency-weighted distributions for TCEM as shown in Figure 2A. Each TCEM in the proteins of the proteomes of Figure 6 was assigned its standardized frequency (σ position on the normal distribution curve in the Human proteome shown in Figure 3A). Then, this TCEM metric was averaged across each protein to give each protein an overall score, essentially a “human-like” score for an entire protein. As an example Figure S8 in Supplemental Material shows how the IgV and human score compare for *B. pseudomallei*. The patterns demonstrate that the high frequency IgV-origin TCEM also indicates an unusually high content of Human proteome motifs.

#### Organism-Specific Motif Signatures

**Figure 8:**
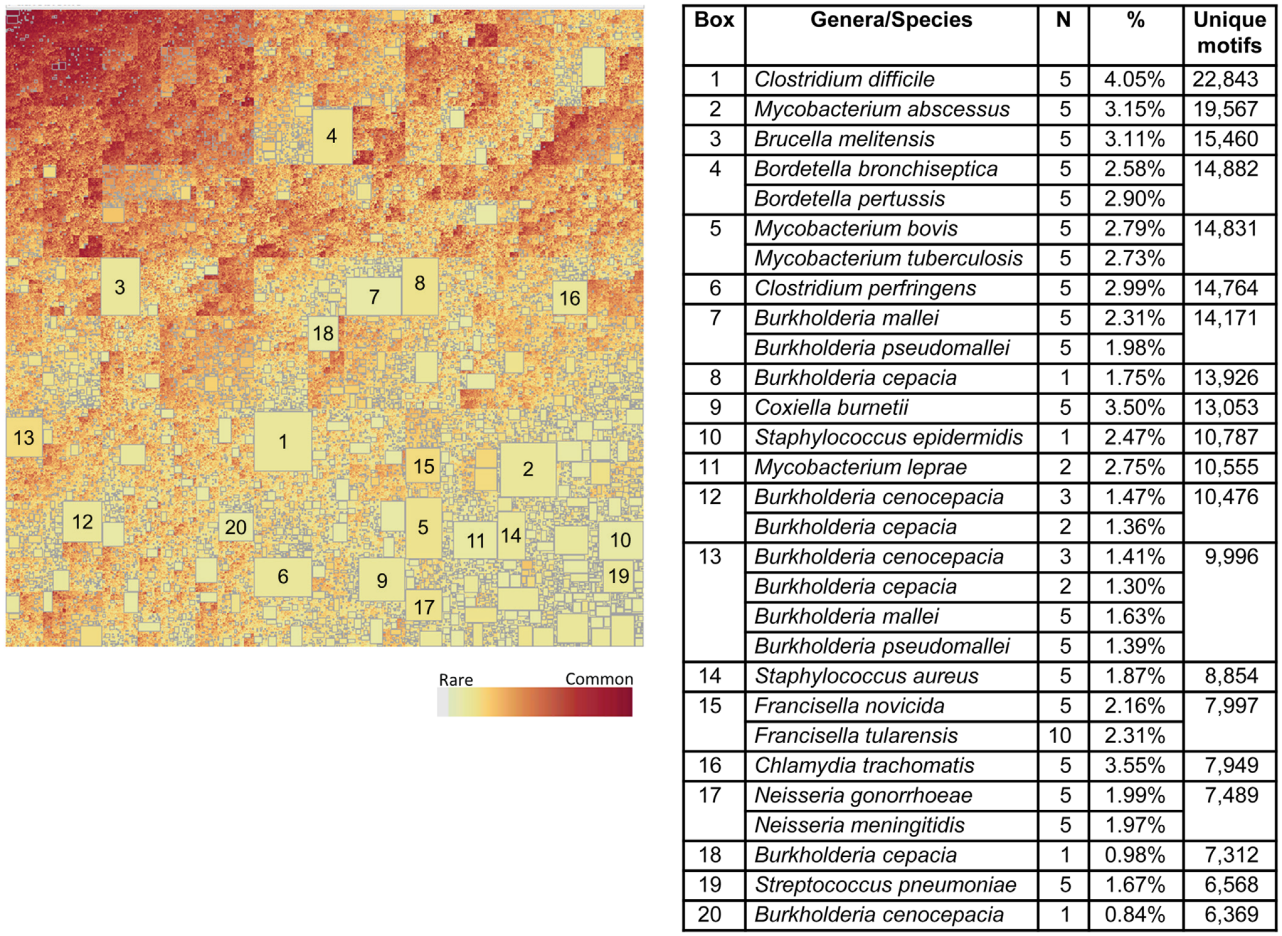
**Treemap of TCEM IIa frequency patterns in the Pathogen proteomes**. Each organism shows a different pattern of occurrence of the possible 20^5^ total motifs. This is a binary absence/presence depiction analogous to a Venn diagram. It comprises 1,540,176 different patterns of 110 million motifs. To construct this map tallies were made of each of the TCEM IIa motifs found in each organism’s proteome. A binary coding scheme was used to score the presence or absence of a particular motif (of 20^5^ total) in a proteome. The size of the rectangle is proportional to the number of motifs. The lightly colored rectangles indicate relatively rare motifs and the dark rectangles indicate common motifs. The large rectangles indicate motifs unique to one or a few species; the organism corresponding to each large rectangle is shown in the accompanying tabulation along with the number of motifs in each of the N proteome and the fraction of the total of each proteome of the organism represented by those motifs. Not visible at this resolution in the upper left hand, darkly colored area, there is a rectangle comprising 615 “universal” motifs that are found in every proteome in this set.

We next addressed the genus-specific occurrence of TCEM within the proteome datasets by using treemapping to examine the intraset differences in presence and absence of motifs. The treemaps each depict the underlying patterns of occurrence over 10^8^ TCEM. The graphics each contains between 1.5 and 2.5 million rectangles. Each rectangle represents a unique TCEM sharing relationship of motifs among bacterial species and is sized to represent the number of motifs within that combination. The treemap for the Pathogen data set is shown in Figure 8. Figure S12 in Supplementary Material shows magnification of a small area of the upper left corner of Figure 8 to show the underlying detail of motif sharing relationships. Strikingly, within the very large Pathogen dataset there are a number of relatively large rectangles visible in Figure 8. Each of the large rectangles contains a motif combination, or signature, that is unique to one or more species of a single genus. For instance, *Streptococcus pneumoniae* (Box 19) has 6,568 TCEM IIa pentamers not found in the other 131 species analyzed. The top 20 in each of the Pathogen proteomes is indicated in the table accompanying Figure 8. Each of these 20 rectangles each represents 0.84–4.05% of the total proteome of an organism. Thus, each organism contains a subset of TCEM that are unique to itself and that in some cases comprises a significant fraction of its entire proteome. There are also sets of motifs that are universally shared or nearly so, shown in dark red boxes in the upper left corner of the treemap. These are the motifs that are found in every bacterial species in the set.

**Figure 9:**
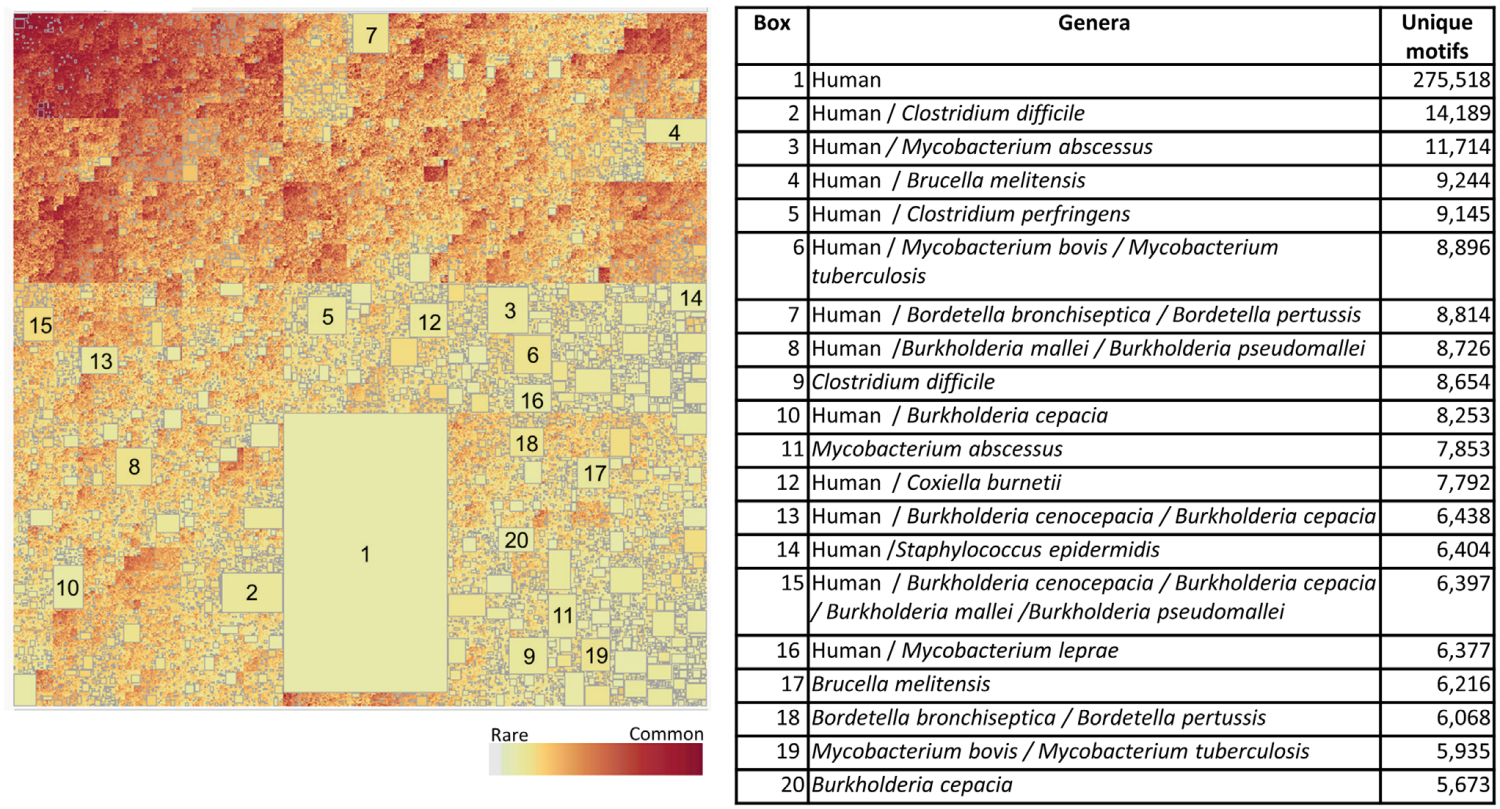
**Treemap of combined TCEM IIa frequency patterns in the Pathogen proteomes and the Human proteome**. The algorithm is the same as used in Figure 7 but in this case the human proteome was also included in the analysis. This shows the inter-relationship between the motifs of the Pathogen and the Human. It comprises 1,585,188 different motif sharing patterns. The accompanying tabulation of the 20 largest boxes indicates the composition depicted in these rectangles. For example, Box 1 shows that there are 275,510 motifs are unique to humans and not found in any of the pathogens whereas Box 2 shows the number of motifs that are found in both the *C difficile* and Human proteomes and indicates that 14,189 of the 22,843 unique Pathogen motifs found in the organism (Figure 7) are also found in the Human proteome. Overall, this shows a remarkable overlap between the T-cell motifs found in the proteins of pathogens and those found in humans.

When the Pathogen set was then combined with the Human proteome it emerges that that nearly every bacterial species has a subset of its genus-specific TCEM that it shares with the Human proteome (Figure 9). This overlap is to be expected based on the general overlaps shown in Table 1. In Figure 9, the TCEMs unique to the Human proteome and not found in any of the 132 proteomes of the Pathogen set are represented in the largest rectangle. The other large rectangles identify motifs shared by Human proteome and one or more Pathogens as indicated in the Table in Figure 9. For example, *Coxiella burnetti* (Box 12) has 7,792 motifs uniquely shared with the Human. A human would obviously not encounter all of the Pathogens simultaneously and the patterns seen when the Human proteome is compared to any one of the Pathogens is itself quite striking. Figure 5B shows the amino acid composition of the 615 motifs shared among Human and both bacterial proteome sets. These motifs favor leucine, glycine, alanine, and isoleucine.

**Figure 10:**
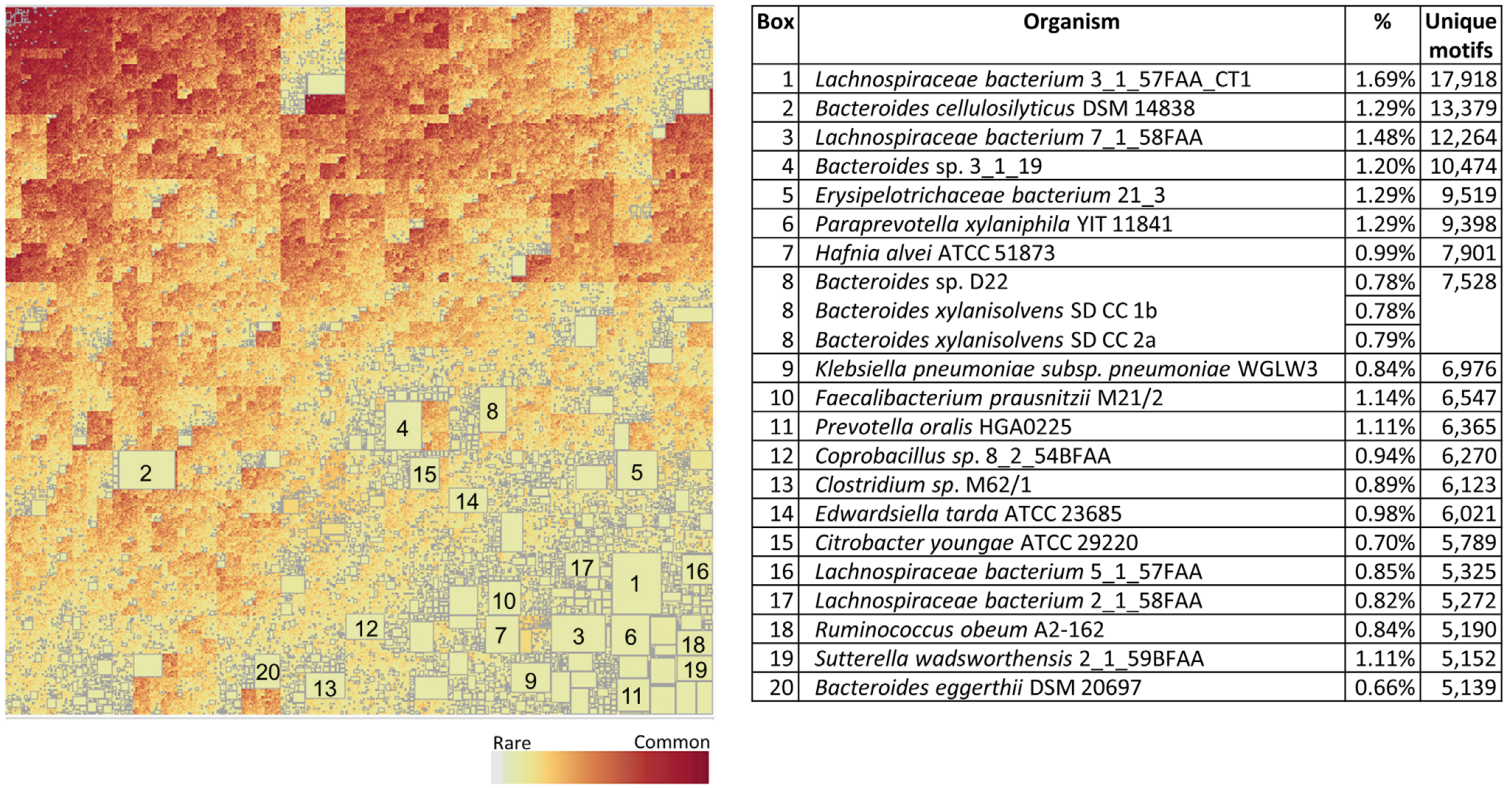
**Treemap of TCEM IIa frequency patterns in the Microbiome proteomes**. See Figure 7 for more complete description. This treemap comprises 2,092,911 different motif sharing patterns.

**Figure 11:**
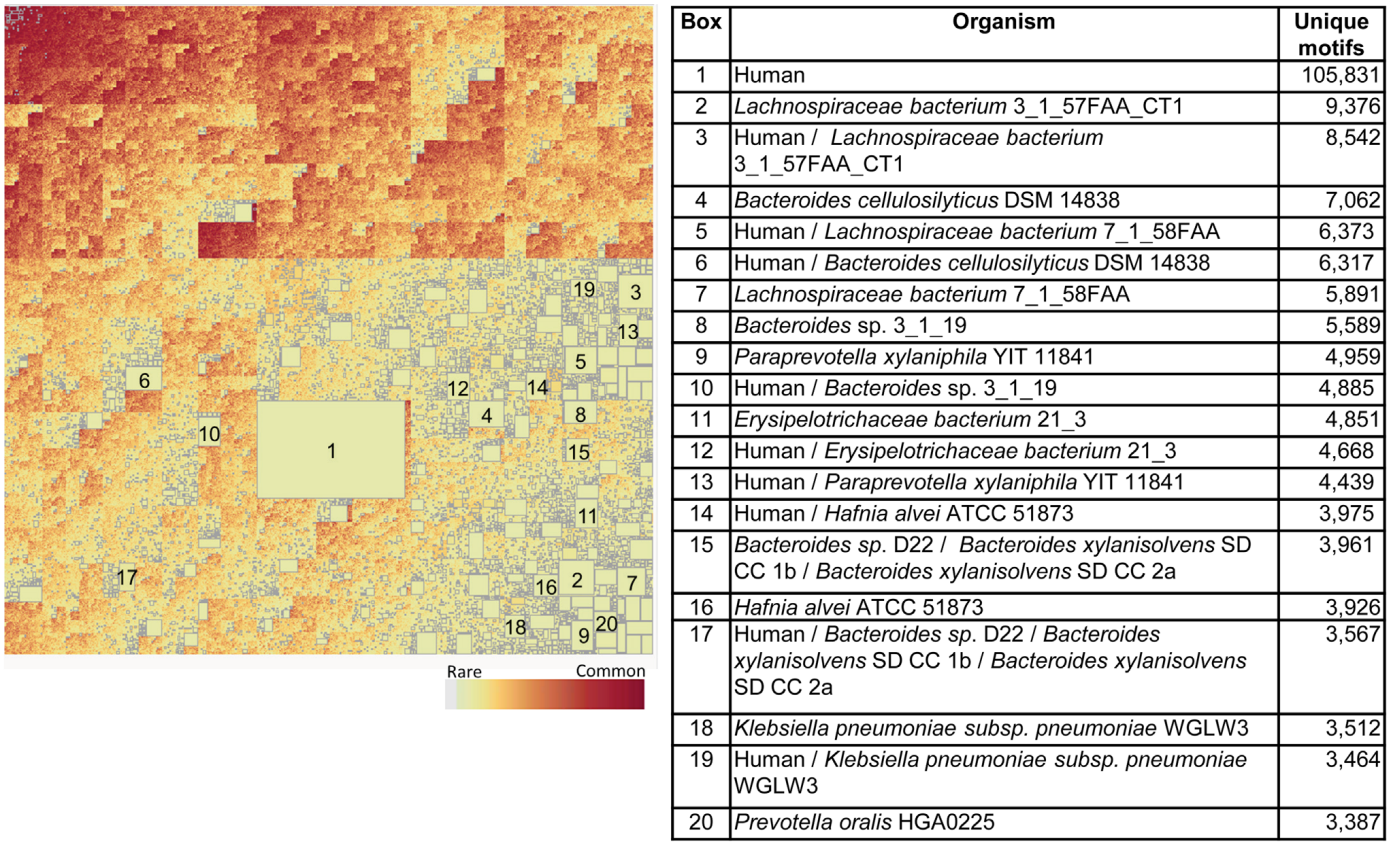
**Intersection between the Human proteome and the Microbiome**. See Figure 9 for more complete description. This treemap comprises 2,144,076 different motif sharing patterns.

Figure 10 shows the corresponding patterns of unique bacterial signatures for the Microbiome bacteria analyzed. Here the unique signatures tend to constitute a smaller fraction of the individual proteomes, from 0.66 to 1.7%, however this may be a reflection of the greater diversity of genera in this dataset. Figure 11 shows the unique patterns of sharing with the Human proteome of TCEM in the Microbiome.

**Figure 12:**
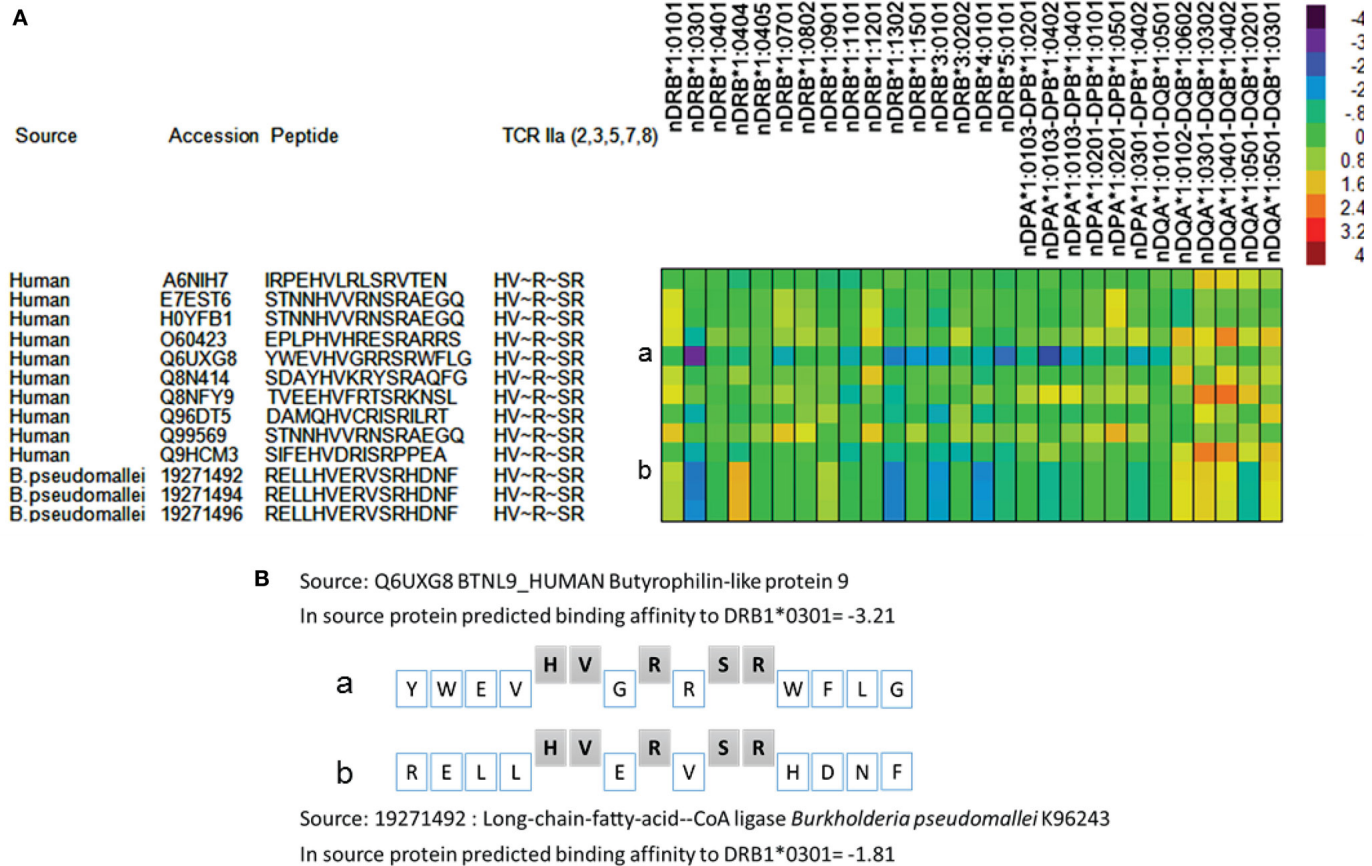
**Differential predicted MHC binding of peptides from human and bacterial proteomes with identical TCEM but different groove exposed motifs**. Examples shown are from Box 8 in Figure 9, which comprises 8,726 total motifs. **(A)** Binding affinities were computed for the MHC II alleles indicated using previously described methods (18) and standardized to zero mean and unit variance within the parent protein. The color scale shows predicted binding in SD units. Source proteins listed by Accession number are listed in full in Table S10 in Supplementary Material. **(B)** Peptides a and b shown in **(A)** are shown with the T-cells exposed amino acids and the groove exposed amino acids identified.

To illustrate how identical TCEM may be associated with multiple different GEMs and thus have different MHC binding affinities in their source protein, a small subset of TCEM shared between Human proteome and *Burkholderia pseudomallei* (Box 8 of Figure 9) were examined. An example of this comparison is provided in Figure 12. Here it is seen that a single TCEM IIa ~~~~HV~R~SR~~~~ was found in 10 different human proteins and 3 different *B. pseudomallei* proteins. The 15-mer context is different in each case and this means the predicted MHC binding for each is different. While DRB1*01:01 binds relatively poorly to all the 15-mers, DRB1*03:01 binds more strongly and in particular when the TCEM is in the context of one human protein 15-mer (YWEVHVGRRSRWFLG) and the three *B. pseudomallei* proteins. While for each allele, the same T-cell would recognize the same TCEM, the cumulative duration and repetition of interaction would depend on the effect of the binding affinity on the peptide dwell time in the pMHC complex, and thus the probability of a productive T-cell encounter occurring.

As shown in Figure 3, TCEM frequencies are log-normally distributed and appear to be the result of random processes. Thus, unique motifs might occur at random anywhere in a proteome. However, on closer inspection a substantial number Pathogen-unique “signature motifs” appear to cluster in individual proteins, and even in relatively contiguous segments of proteins. A majority of the unique motifs represented in the larger boxes in the treemaps (Figure 8) are found in segments of only 50–100 different proteins in each proteome.

## Discussion

In the present study, we compare two diverse sets of composite bacterial proteomes, which coexist with the human proteome, by using the recognition patterns of the cellular immune system. We also compare the occurrence of the same recognition patterns in IgV and the human proteome. Genomic comparisons generally focus on analysis of single nucleotide or amino acid changes. By opting to use the TCEMs as the comparator, we are in effect looking how the proteins in the organisms are recognized by the T-cells as the initiator of any selective pressure by the cellular immune system.

A central challenge in immunology is to understand how self is distinguished from non-self. What emerges from our analysis is that, at least for the T-cell response, this concept is a considerable oversimplification of a complex signaling system. Indeed, what we observe in the present analysis is that what may be uniquely human is, at best, only a very small subset of the possible T-cell recognition signals. When viewed as TCEM pentamers recognized by T-cells in contact pattern IIa and comparing the human proteome to a range of pathogenic and commensal bacteria, only 1.9% of the possible pentamers are unique to humans. If we consider all three contact pattern of T-cell recognition (TCEM I, IIa and IIb) then the truly unique peptides may be the multiple of these metrics and much smaller. The Microbiome dataset analyzed here is only a small subset of the total gastrointestinal microbiome. An individual human would not be exposed to all of the Pathogens simultaneously. However, over time, the larger the number of bacteria that are encountered, the more closely the number of TCEM unique to humans approaches zero. The organism-specific patterns would be expected to play a role in the overall recognition. Although no single TCEM that an individual T-cell receptor binds apparently defines self, the T-cell repertoire as a whole is capable of uniquely recognizing the overall composite patterns.

The TCEM does not function alone in determining the outcome of a T-cell–peptide interaction, but is recognized within the context of the surrounding MHC histotope. An individual host has a limited set of MHC alleles, which can present a peptide and thus form the surrounding histotope surface. Furthermore, the binding affinity of the inner face of the MHC-bound peptide, the GEM, determines the dwell time of the peptide in the MHC groove and hence the probability of productive T-cell:pMHC encounter, the duration of any T-cell interaction, or how many times it is repeated. Here again an individual’s HLA alleles are determinants of the T-cell response. Thus, both “faces” of the peptide, T-cell exposed and groove exposed, modulate the overall outcome in the context of the host’s immunogenetics (19, 20). One example of this is provided in Figure 12.

The diversity in possible GEMs is also vast. In the case of MHC I, the GEMs comprise 20^4^ potential combinations (assuming a 9-mer peptide), and for MHC II a further 20^10^ (assuming a 15-mer) combinations exist. These 20^10^ variants in GEM are multiplied by the potential 3.2 million different TCEMs to arrive at the maximum number of potential specific interactions of peptides with T-cells in each of the three contact patterns. GEMs determine on-off rate, or duration, of peptide MHC binding. However, it is the TCEM, and the frequency of its occurrence, which determines the size of the cognate T-cell population responding to the peptide and which can hence contribute to T-cell repertoire phenotypes and the balance of immune stimulation or suppression (21, 22).

Common TCEM, like those found repeatedly in IgV, provide for a higher frequency of interaction with cognate T-cells. Higher signal strength generated through a combination high frequency pMHC:T-cell contact and longer interaction dwell time favors emergence of a Treg phenotype, through a combination of Foxp3 induction (23) and epigenetic changes in the T-cells (24–26). Higher affinity MHC binding of the peptide GEM ensures a longer dwell time and thus more opportunity for interactions at the T-cell exposed face. Implicitly, high frequency TCEMs may build up a larger cognate T-cell population. So provided they also occur in peptides which bind well to the MHC, high frequency TCEMs will therefore be expected to generate more Tregs. The balance of Treg and T-effectors is modulated by IL2 secretion by T-effectors and binding and removal of IL2 by Treg (27–29). Local binding of IL2 will accelerate Treg proliferation, suppressing effector responses in the vicinity and potentially leading to greater numbers of residual cells of the same specificity available for future interaction (30). Such a feedback loop driven by the frequency of TCEM appearance is consistent with a form of quorum sensing among T-cells (31). It also indicates that down-regulation and homeostasis is likely the norm driven by common TCEM. Conversely, the lower frequency motifs are likely to be more important in discriminating between self and non-self and in generating an upregulated immune response.

A further consideration is the abundance of the protein in which a TCEM is located, how frequently it is transcribed and catabolized, and its accessibility to antigen presenting cells. In the case of bacterial proteomes, these factors will change either as different proteins are expressed with the stage of infection or with changes in the microbial ecology of the microbiome. Interestingly, this may make the modulating role of immunoglobulin-derived peptides relatively more important as immunoglobulins are constantly accessible, readily processed, and presented by B cells.

While we have interpreted the exposure of amino acids in TCEMs in three simple contact patterns based on the structural analysis of Rudolph et al. (2), flexibility of peptides may accommodate some bulging and twisting of MHC-bound peptides to create some variability in the TCEM exposure (32) and so we expect that the interaction will be much more nuanced. Additionally other studies have examined the relative importance of each amino acid in the binding groove (33). A further compounding factor is that there may be multiple clonal lines of cognate T-cells for any unique TCEM; there is not a one-to-one correspondence of TCEM and T-cell receptor and a T-cell receptor may bind with more than one TCEM-MHC combination, through recognition of physicochemical near neighbors (16, 17).

### Comparative Overlaps in Motifs Among Proteomes Analyzed

The bacteria selected for inclusion in the Pathogen set comprised human pathogens, which are generally directly transmitted, not vector borne. With exception of the *Clostridium spp*, we excluded genera that are typically fecal-oral transmission. This was intended to maintain a clear distinction from the array of bacteria included in the gastrointestinal Microbiome group. Similarly, we opted to include *Escherichia spp* in the Microbiome set. Such decisions about opportunist pathogens certainly affected the degree of overlap we observed, but do not detract from the overall conclusions. It is noteworthy that *E. coli* is an outlier among the Microbiome set in Figure 7 with respect to its IgV TCEM content. This is consistent with the prior observations that *E. coli* contains a number of immunoglobulin domains (34).

As bacteria are constantly evolving at different rates, and microbiome composition is affected by external factors, the isolates from which we analyzed sequences simply represent a cross sectional sample or “snapshot” of a dynamic process. Had different bacterial isolates been incorporated into the analysis, results would have differed in detail, but likely not the overall conclusions. Similarly, by selecting the gastrointestinal microbiome, we do not underestimate that the microbiota of other mucosal surfaces exercise a similar relationship with the human proteome and IgV and undoubtedly also influence immune modulation (35).

In the analysis conducted, we demonstrate that there are different and distinctive patterns of TCEM occurrence in the three proteome groups. Interestingly, the Human proteome utilizes only about three quarters of the possible pentamers and has a relatively small set of 59,256 unique motifs not found in the Microbiome and Pathogen proteomes analyzed. Both bacterial proteome sets we examine overlap extensively with the Human proteome. Slightly more overlap occurs with the resident gastrointestinal Microbiome perhaps indicative of a longer homeostatic coexistence.

This high degree of TCEM commonality underscores the impossibility of central tolerance to all self-antigens (36, 37). Rather, it argues for functional modulation of peripheral T-cell responses to specific TCEM along a spectrum of Treg to T-effector, enabled by T-cell plasticity of phenotype (38, 39), and the localization of responses in response to mucosal microbiome or to infection (40–42).

While there appears to be no overriding requirement for any given motif driven by basic protein structure, there is a fairly large set of almost 121,683 motifs, which are absent from all three proteome groups. These “forbidden” motifs have amino acid compositions in common that are high in cysteine, histidine, methionine and tryptophan (Figure 5A). The side chains of these amino acids each have physicochemical properties that make it unlikely for them to be in close proximity to each other, which may bias against their use. Conversely, there is a group of motifs that are a relatively smaller “universal set” found in all organisms. Amino acid compositions of these TCEM showed a high content of the hydrophobic amino acids known to be commonly found in membrane-spanning helices.

### Motif Patterns in Immunoglobulins

We show that the diversity of peptides generated within immunoglobulin variable regions is broadly representative of the TCEM in all three proteome datasets and our IgV dataset comprises a stratified random sample across the array of all possible TCEM. We see that our database of 56,000 IgV sequences provides a balanced random coverage for both the human proteome and the two bacterial proteome sets we used in our analysis, and even represents the “forbidden” motifs never used in the three proteome groups. Thus, the somatic mutational process that generates immunoglobulin variable regions is evidently capable of generating any possible 9-mer or 15-mer peptide. As the dataset of immunoglobulins analyzed is only about 1% of the full complement of IgV that any individual would carry (12), it appears that the diversity of TCEM within IgV may be able to stimulate and maintain a T-cell repertoire that is capable of responding to all potential T-cell immunogens. This supports the hypothesis that the frequency of motif occurrence in the IgV may modulate the T-cell repertoire in the face of selective pressure and assist in maintaining homeostasis and memory (1).

Immunoglobulins are unique compared to the rest of the human proteome in their immense diversity and in their turnover rate. A human carries about 3–9 × 10^6^ unique B-cell clonotypes (12) yielding variable regions which, combining both light and heavy chains, each have approximately 260 unique sequential peptides. In aggregate, therefore, the immunoglobulin of an individual human comprises a billion or more unique peptides yielding TCEMs in each contact pattern. Moreover, the lifespan of a B-cell is estimated to be of 30–60 days (43) and each immunoglobulin about 14 days (44), so there is considerable turnover. At a serum concentration of about 11 g/l the diverse mass of IgV constitutes about 75 g in an adult human (45, 46). In contrast, the remainder of the human proteome comprises approximately 20,000 proteins averaging about 384 amino acids apiece, or about 7 million unique peptides and has a slower rate of turnover. Thus, immunoglobulins constitute a massive, dynamic protein pool with enormous peptide diversity available throughout the body.

The broad representation of motifs within the IgV with a range of frequency-of-occurrence of about 2^16^ and which are being consistently presented to T-cells (6), also implies that at any time the T-cell repertoire contains a gradation in frequency of cognate T-cells ready to respond to a fraction of the Pathogen or Microbiome motifs whenever these are first encountered. These may constitute a “starter set” of first responders, which can expand in size and which can be supplemented by respondents to the other motifs. This response would be supplemented by near neighbor matches, in which four of five amino acids generate a near fit (16, 17). The sharing of the same T-cell recognition motifs by the human proteome, IgV, and in the Pathogen sets accounts for the observations by others that naïve T-cells sampled from cord blood have the innate ability to recognize pathogens (47, 48). While most observations on cord blood have focused on viral pathogens there is no reason to think they would be more diverse in TCEM usage than bacteria. It is also consistent with the role of the microbiota in maintaining immune homeostasis (35).

The frequency distribution of motif occurrence in each of the three proteome sets is very similar and is log-normal. In contrast, the IgV-mutated set shows a Pareto distribution strongly biased toward the more common motifs (Figure 4). Although any peptide can be generated by the IgV mutational process, within the IgV data set we examined, half of all the possible TCEM motifs reappear at least once in every 1024 B-cell clonal products (1). This means there is a greater degree of proteome overlap by the high frequency IgV motifs and a lesser overlap by the rare ones. The implication is that more common IgV TCEMs will likely engender larger T-cell populations, more memory, and more Tregs.

Not only is the somatic mutation process capable of generating a highly diverse set of TCEM, the appearance of TCEM in the IgV constitutes a stratified random sample of the TCEM distribution in the Human proteome and the bacterial proteomes. This implies that the TCEM frequency distribution observed in IgV can likewise be used as a means of characterization of TCEM in proteins from outside of the human proteome.

### Patterns Within the Pathogen Set

Members of the Pathogen set show clear differences in IgV-like motif content in their constituent proteins (Figure 6). Perhaps the most striking feature of this pattern is that two genera, *Burkholderia* and *Mycobacterium*, both intracellular organisms that cause chronic diseases, stand out as having many proteins that contain motifs used at a high frequency in the IgV. In some proteins the IgV matched TCEM content is over 50% of the total TCEM motifs. These two genera also have a high level of uniquely shared motifs with the Human proteome exclusive of IgV (Figures 8 and 9). Selective advantage may be provided by the camouflage of using very common and likely immunosuppressive motifs. This is consistent with findings reported for *Mycobacteria* (49, 50). Further, in some pathogens, including *Mycobacteria*, we noted a higher distribution of IgV motifs in proteins that are upregulated early in infection (data not shown), indicating that the spectrum of overlap and immune recognition may change through the infective cycle.

A second striking observation is that, even with the limitation of 3.2 million TCEM motifs, certain subsets of a few thousand motifs were unique to particular bacteria, sometimes down to the species level. This pattern is maintained when the motifs in common with the human proteome are identified. While this pattern will undoubtedly become more diffuse as more organisms are added to the analysis set, it indicates that there are specific signatures that could serve as targets for interventions.

Both these genus- and species-specific patterns point to possible different mechanisms of immune evasion arising from evolutionary pressure. For instance, while *Burkholderia* and *Mycobacterium* share many motifs with IgV and Human proteome and also have fairly large sets of species specific motifs, *Francisella* is conspicuously absent from the larger clusters of unique signatures seen in Figures 8 and 9 and has a lower overall IgV TCEM content (Figure 6). It is not obvious what the evolutionary forces are which give rise to such signature patterns, but their existence suggests an important further line of inquiry toward better understanding of immune evasion and pathogenesis and to provide critical information for vaccine design.

The boxes in Figures 9 and 11, showing the complexity of TCEM overlap between Pathogen or Microbiome organisms with the Human proteome, indicate the extent to which a T-cell responsive to a bacteria may also respond to a human protein. Further examination of this overlap may shed light on how infections or alterations in commensal balance can trigger the onset of autoimmune diseases.

The limitation of 3.2 million TCEMs in each frame accounts for the widely observed T-cell polyspecificity (51–53) and the overall physical limitations on T-cell repertoire size deduced by Mason (3). The motifs within the total possible array of 3.2 million and, as we indicate here, a somewhat smaller actual utilized repertoire, have to enable recognition of all peptides of self and non-self origin. Unique recognition, and thus initial response as well as recall, depends on the combination of signals, not on a single unique motif, and thus on the aggregate response of multiple T-cell clonal populations.

## Conclusion

The analysis of proteomes based on the TCEM contact patterns provides insight into the immune recognition of different classes of organism. Overall, the approach we have used to analyze commonalities and differences provides a new perspective on the discrimination between self and non-self, underscoring that there is very little uniqueness to the individual TCEMs present in the human proteome. Thus, distinguishing self and not self is dependent on a pattern comprised of many TCEM and which includes the relative frequency of each. This analytical approach can be applied more broadly. Any protein, proteome, or collection of proteomes, or mutated neoantigens, can be characterized by their TCEM pentamer content and frequency. This can provide insights and new experimental approaches to autoimmunity, allergies, and other immunopathologies. In the case of the Pathogen set, the TCEM analysis indicates clear differences in motif content between proteins of different genera and species and provides clues to both immune evasion and strategies for intervention, including vaccine design. The breadth and distinct frequency patterns of the immunoglobulin-derived peptides again suggests a role of immunoglobulins in maintaining a broadly responsive T-cell repertoire and memory.

## Conflict of Interest Statement

Both authors are employees and equity holders in ioGenetics LLC, the parent company of EigenBio LLC.

## Footnotes

^1^www.uniprot.org

^2^www.hmpdacc.org/HMRGD

^3^www.patricbrc.org

## References

[1] Bremel RD Homan EJ. Frequency patterns of T-cell exposed amino acid motifs in immunoglobulin heavy chain peptides presented by MHCs. Front Immunol (2014) 5:541. 10.3389/fimmu.2014.00541 25389426 PMC4211557

[2] Rudolph MG Stanfield RL Wilson IA. How TCRs bind MHCs, peptides, and coreceptors. Annu Rev Immunol (2006) 24:419–66. 10.1146/annurev.immunol.23.021704.115658 16551255

[3] Mason D. A very high level of crossreactivity is an essential feature of the T-cell receptor. Immunol Today (1998) 19(9):395–404. 9745202 10.1016/s0167-5699(98)01299-7

[4] Troy AE Shen H. Cutting edge: homeostatic proliferation of peripheral T lymphocytes is regulated by clonal competition. J Immunol (2003) 170(2):672–6. 10.4049/jimmunol.170.2.672 12517927

[5] Sy MS Benacerraf B. Suppressor T cells, immunoglobulin and Igh restriction. Immunol Rev (1988) 101:133–48. 10.1111/j.1600-065X.1988.tb00735.x 2965093

[6] Weiss S Bogen B. B-lymphoma cells process and present their endogenous immunoglobulin to major histocompatibility complex-restricted T cells. Proc Natl Acad Sci U S A (1989) 86(1):282–6. 10.1073/pnas.86.1.282 2492101 PMC286448

[7] Chakrabarti D Ghosh SK. Induction of syngeneic cytotoxic T lymphocytes against a B cell tumor. III. MHC class I-restricted CTL recognizes the processed form(s) of idiotype. Cell Immunol (1992) 144(2):455–64. 10.1016/0008-8749(92)90259-R 1394454

[8] Rudensky A Preston-Hurlburt P Al-Ramadi BK Rothbard J Janeway CA Jr. Truncation variants of peptides isolated from MHC class II molecules suggest sequence motifs. Nature (1992) 359(6394):429–31. 10.1038/359429a0 1328884

[9] Bogen B Ruffini P. Review: to what extent are T cells tolerant to immunoglobulin variable regions? Scand J Immunol (2009) 70(6):526–30. 10.1111/j.1365-3083.2009.02340.x 19906193

[10] Joao C Ogle BM Gay-Rabinstein C Platt JL Cascalho M. B cell-dependent TCR diversification. J Immunol (2004) 172(8):4709–16. 10.4049/jimmunol.172.8.4709 15067046

[11] AbuAttieh M Bender D Liu E Wettstein P Platt JL Cascalho M. Affinity maturation of antibodies requires integrity of the adult thymus. Eur J Immunol (2012) 42(2):500–10. 10.1002/eji.201141889 22105515 PMC4855521

[12] Arnaout R Lee W Cahill P Honan T Sparrow T Weiand M High-resolution description of antibody heavy-chain repertoires in humans. PLoS One (2011) 6(8):e22365. 10.1371/journal.pone.0022365 21829618 PMC3150326

[13] UniProt Consortium. UniProt: a hub for protein information. Nucleic Acids Res (2015) 43(Database issue):D204–12. 10.1093/nar/gku989 25348405 PMC4384041

[14] Wattam AR Abraham D Dalay O Disz TL Driscoll T Gabbard JL PATRIC, the bacterial bioinformatics database and analysis resource. Nucleic Acids Res (2014) 42(Database issue):D581–91. 10.1093/nar/gkt1099 24225323 PMC3965095

[15] Lilliefors HW On the Kolmogorov-Smirnov test for normality with mean and variance unknown. J Am Stat Assoc (1967) 62:399–402. 10.1080/01621459.1967.10482916

[16] Petrova GV Gorski J. Cross-reactive responses to modified M1(5)(8)-(6)(6) peptides by CD8(+) T cells that use noncanonical BV genes can describe unknown repertoires. Eur J Immunol (2012) 42(11):3001–8. 10.1002/eji.201242596 22865108 PMC3817827

[17] Nelson RW Beisang D Tubo NJ Dileepan T Wiesner DL Nielsen K T cell receptor cross-reactivity between similar foreign and self peptides influences naive cell population size and autoimmunity. Immunity (2015) 42(1):95–107. 10.1016/j.immuni.2014.12.022 25601203 PMC4355167

[18] Bremel RD Homan EJ. An integrated approach to epitope analysis II: a system for proteomic-scale prediction of immunological characteristics. Immunome Res (2010) 6(1):8. 10.1186/1745-7580-6-8 21044290 PMC2991286

[19] Moise L Gutierrez AH Bailey-Kellogg C Terry F Leng Q Abdel Hady KM The two-faced T cell epitope: examining the host-microbe interface with JanusMatrix. Hum Vaccin Immunother (2013) 9(7):1577–86. 10.4161/hv.24615 23584251 PMC3974887

[20] Birnbaum ME Mendoza JL Sethi DK Dong S Glanville J Dobbins J Deconstructing the peptide-MHC specificity of T cell recognition. Cell (2014) 157(5):1073–87. 10.1016/j.cell.2014.03.047 24855945 PMC4071348

[21] Gett AV Sallusto F Lanzavecchia A Geginat J. T cell fitness determined by signal strength. Nat Immunol (2003) 4(4):355–60. 10.1038/ni908 12640450

[22] Jenkins MK Moon JJ. The role of naive T cell precursor frequency and recruitment in dictating immune response magnitude. J Immunol (2012) 188(9):4135–40. 10.4049/jimmunol.1102661 22517866 PMC3334329

[23] Josefowicz SZ Lu LF Rudensky AY. Regulatory T cells: mechanisms of differentiation and function. Annu Rev Immunol (2012) 30:531–64. 10.1146/annurev.immunol.25.022106.141623 22224781 PMC6066374

[24] Ohkura N Hamaguchi M Morikawa H Sugimura K Tanaka A Ito Y T cell receptor stimulation-induced epigenetic changes and Foxp3 expression are independent and complementary events required for Treg cell development. Immunity (2012) 37(5):785–99. 10.1016/j.immuni.2012.09.010 23123060

[25] Vahl JC Drees C Heger K Heink S Fischer JC Nedjic J Continuous T cell receptor signals maintain a functional regulatory T cell pool. Immunity (2014) 41(5):722–36. 10.1016/j.immuni.2014.10.012 25464853

[26] Liu B Chen W Evavold BD Zhu C. Accumulation of dynamic catch bonds between TCR and agonist peptide-MHC triggers T cell signaling. Cell (2014) 157(2):357–68. 10.1016/j.cell.2014.02.053 24725404 PMC4123688

[27] Sakaguchi S Sakaguchi N Shimizu J Yamazaki S Sakihama T Itoh M Immunologic tolerance maintained by CD25+ CD4+ regulatory T cells: their common role in controlling autoimmunity, tumor immunity, and transplantation tolerance. Immunol Rev (2001) 182:18–32. 10.1034/j.1600-065X.2001.1820102.x 11722621

[28] Hofer T Krichevsky O Altan-Bonnet G. Competition for IL-2 between regulatory and effector T cells to chisel immune responses. Front Immunol (2012) 3:268. 10.3389/fimmu.2012.00268 22973270 PMC3433682

[29] Smigiel KS Srivastava S Stolley JM Campbell DJ. Regulatory T-cell homeostasis: steady-state maintenance and modulation during inflammation. Immunol Rev (2014) 259(1):40–59. 10.1111/imr.12170 24712458 PMC4083836

[30] Leignadier J Labrecque N. Epitope density influences CD8+ memory T cell differentiation. PLoS One (2010) 5(10):e13740. 10.1371/journal.pone.0013740 21060788 PMC2966420

[31] Almeida AR Amado IF Reynolds J Berges J Lythe G Molina-Paris C Quorum-sensing in CD4(+) T cell homeostasis: a hypothesis and a model. Front Immunol (2012) 3:125. 10.3389/fimmu.2012.00125 22654881 PMC3360200

[32] Petrova G Ferrante A Gorski J. Cross-reactivity of T cells and its role in the immune system. Crit Rev Immunol (2012) 32(4):349–72. 10.1615/CritRevImmunol.v32.i4.50 23237510 PMC3595599

[33] Painter CA Stern LJ. Conformational variation in structures of classical and non-classical MHCII proteins and functional implications. Immunol Rev (2012) 250(1):144–57. 10.1111/imr.12003 23046127 PMC3471379

[34] Bodelon G Palomino C Fernandez LA. Immunoglobulin domains in *Escherichia coli* and other enterobacteria: from pathogenesis to applications in antibody technologies. FEMS Microbiol Rev (2013) 37(2):204–50. 10.1111/j.1574-6976.2012.00347.x 22724448

[35] Belkaid Y Hand TW Role of the microbiota in immunity and inflammation. Cell (2014) 157(1):121–41. 10.1016/j.cell.2014.03.011 24679531 PMC4056765

[36] Attridge K Walker LS. Homeostasis and function of regulatory T cells (Tregs) in vivo: lessons from TCR-transgenic Tregs. Immunol Rev (2014) 259(1):23–39. 10.1111/imr.12165 24712457 PMC4237543

[37] Caton AJ Kropf E Simons DM Aitken M Weissler KA Jordan MS. Strength of TCR signal from self-peptide modulates autoreactive thymocyte deletion and Foxp3(+) Treg-cell formation. Eur J Immunol (2014) 44(3):785–93. 10.1002/eji.201343767 24307208 PMC3959276

[38] Bluestone JA Mackay CR O’Shea JJ Stockinger B. The functional plasticity of T cell subsets. Nat Rev Immunol (2009) 9(11):811–6. 10.1038/nri2654 19809471 PMC3075537

[39] Sawant DV Vignali DA. Once a Treg, always a Treg? Immunol Rev (2014) 259(1):173–91. 10.1111/imr.12173 24712466 PMC4008876

[40] Hsieh CS Zheng Y Liang Y Fontenot JD Rudensky AY. An intersection between the self-reactive regulatory and nonregulatory T cell receptor repertoires. Nat Immunol (2006) 7(4):401–10. 10.1038/ni1318 16532000

[41] Barnes MJ Griseri T Johnson AM Young W Powrie F Izcue A. CTLA-4 promotes Foxp3 induction and regulatory T cell accumulation in the intestinal lamina propria. Mucosal Immunol (2013) 6(2):324–34. 10.1038/mi.2012.75 22910217 PMC3574974

[42] Sathaliyawala T Kubota M Yudanin N Turner D Camp P Thome JJ Distribution and compartmentalization of human circulating and tissue-resident memory T cell subsets. Immunity (2013) 38(1):187–97. 10.1016/j.immuni.2012.09.020 23260195 PMC3557604

[43] Fulcher DA Basten A. B cell life span: a review. Immunol Cell Biol (1997) 75(5):446–55. 10.1038/icb.1997.69 9429891

[44] Bonilla FA. Pharmacokinetics of immunoglobulin administered via intravenous or subcutaneous routes. Immunol Allergy Clin North Am (2008) 28(4):803–19. 10.1016/j.iac.2008.06.006 18940575

[45] Dati F Schumann G Thomas L Aguzzi F Baudner S Bienvenu J Consensus of a group of professional societies and diagnostic companies on guidelines for interim reference ranges for 14 proteins in serum based on the standardization against the IFCC/BCR/CAP Reference Material (CRM 470). International Federation of Clinical Chemistry. Community Bureau of Reference of the Commission of the European Communities. College of American Pathologists. Eur J Clin Chem Clin Biochem (1996) 34(6):517–20. 8831057

[46] Gonzalez-Quintela A Alende R Gude F Campos J Rey J Meijide LM Serum levels of immunoglobulins (IgG, IgA, IgM) in a general adult population and their relationship with alcohol consumption, smoking and common metabolic abnormalities. Clin Exp Immunol (2008) 151(1):42–50. 10.1111/j.1365-2249.2007.03545.x 18005364 PMC2276914

[47] Su LF Kidd BA Han A Kotzin JJ Davis MM. Virus-specific CD4(+) memory-phenotype T cells are abundant in unexposed adults. Immunity (2013) 38(2):373–83. 10.1016/j.immuni.2012.10.021 23395677 PMC3626102

[48] Neller MA Ladell K McLaren JE Matthews KK Gostick E Pentier JM Naive CD8 T-cell precursors display structured TCR repertoires and composite antigen-driven selection dynamics. Immunol Cell Biol (2015) 93(7):625–33. 10.1038/icb.2015.17 25801351 PMC4533101

[49] Boer MC Joosten SA Ottenhoff TH. Regulatory T-cells at the interface between human host and pathogens in infectious diseases and vaccination. Front Immunol (2015) 6:217. 10.3389/fimmu.2015.00217 26029205 PMC4426762

[50] Boer MC van Meijgaarden KE Joosten SA Ottenhoff TH. CD8+ regulatory T cells, and not CD4+ T cells, dominate suppressive phenotype and function after in vitro live *Mycobacterium bovis*-BCG activation of human cells. PLoS One (2014) 9(4):e94192. 10.1371/journal.pone.0094192 24714620 PMC3979753

[51] Sewell AK. Why must T cells be cross-reactive? Nat Rev Immunol (2012) 12(9):669–77. 10.1038/nri3279 22918468 PMC7097784

[52] Wooldridge L Ekeruche-Makinde J van den Berg HA Skowera A Miles JJ Tan MP A single autoimmune T cell receptor recognizes more than a million different peptides. J Biol Chem (2012) 287(2):1168–77. 10.1074/jbc.M111.289488 22102287 PMC3256900

[53] Wucherpfennig KW Allen PM Celada F Cohen IR De Boer R Garcia KC Polyspecificity of T cell and B cell receptor recognition. Semin Immunol (2007) 19(4):216–24. 10.1016/j.smim.2007.02.012 17398114 PMC2034306

