# Supplementary material for: Extensive T-Cell Epitope Repertoire Sharing among Human Proteome, Gastrointestinal Microbiome, and Pathogenic Bacteria: Implications for the Definition of Self
Source: Front Immunol. 2015 Oct 22;6:538. doi: 10.3389/fimmu.2015.00538 (PMC4617169; doi:10.3389/fimmu.2015.00538)

Supplementary Material

Extensive T-cell epitope repertoire sharing among human proteome, gastrointestinal microbiome, and pathogenic bacteria: Implications for the definition of self

Robert D. Bremel*, E. Jane Homan
*** Correspondence:** Corresponding Author: robert_bremel@eigenbio.com

Supplemental Tables
Supplemental Figures

Supplemental Table 1 – Pathogen and Gastrointestinal Microbiome proteomes included in analysis.

Complete proteomes of the following organisms were downloaded from PATRIC. Isolate identifications shown are as listed in PATRIC

**Pathogens**
Bordetella bronchiseptica 2371640
Bordetella bronchiseptica 253
Bordetella bronchiseptica D445
Bordetella bronchiseptica RB50
Bordetella bronchiseptica SEAT0006
Bordetella pertussis B1920
Bordetella pertussis Bp H897
Bordetella pertussis Bp SEAT 0004
Bordetella pertussis CHLA15
Bordetella pertussis Tohama I
Brucella melitensis 043
Brucella melitensis ATCC 23457
Brucella melitensis B115
Brucella melitensis M5
Brucella melitensis S66
Burkholderia cenocepacia AU 1054
Burkholderia cenocepacia H111
Burkholderia cenocepacia KC-01
Burkholderia cepacia ATCC 25416
Burkholderia cepacia Bu72
Burkholderia mallei ATCC 10399
Burkholderia mallei ATCC 23344
Burkholderia mallei FMH
Burkholderia mallei JHU
Burkholderia mallei NCTC 10247
Burkholderia pseudomallei 1026b
Burkholderia pseudomallei 1106a
Burkholderia pseudomallei 1106b
Burkholderia pseudomallei K96243
Burkholderia pseudomallei MSHR305
Chlamydia trachomatis A363
Chlamydia trachomatis Ds2923
Chlamydia trachomatis FSW5
Chlamydia trachomatis Jali20
Chlamydia trachomatis Sweden2
Clostridium difficile ATCC 43255
Clostridium difficile ATCC 9689
Clostridium difficile CD165

Clostridium difficile DA00212
Clostridium difficile Y270
Clostridium perfringens ATCC 13124
Clostridium perfringens CPE str F4969
Clostridium perfringens E str JGS1987
Clostridium perfringens JJC
Clostridium perfringens str 13
Coxiella burnetii Cb185
Coxiella burnetii Dugway 5J108-111
Coxiella burnetii RSA 331
Coxiella burnetii RSA 493
Coxiella burnetii Z3055
Francisella novicida FTE
Francisella novicida FTG
Francisella novicida GA99-3548
Francisella novicida GA99-3549
Francisella novicida U112
Francisella tularensis subsp holarctica
Francisella tularensis subsp holarctica 257
Francisella tularensis subsp holarctica F92
Francisella tularensis subsp holarctica FSC022
Francisella tularensis subsp holarctica FSC200
Francisella tularensis subsp tularensis FSC033
Francisella tularensis subsp tularensis FSC198
Francisella tularensis subsp tularensis MA00-2987
Francisella tularensis subsp tularensis NE061598
Francisella tularensis subsp tularensis SCHU S4
Mycobacterium abscessus 4S-0303
Mycobacterium abscessus 5S-1212
Mycobacterium abscessus 6G-0212
Mycobacterium abscessus M156
Mycobacterium abscessus V06705
Mycobacterium bovis BCG str ATCC 35733
Mycobacterium bovis BCG str ATCC 35740
Mycobacterium bovis BCG str ATCC 35743
Mycobacterium bovis BCG str Glaxo
Mycobacterium bovis BCG str Pasteur 1173P2
Mycobacterium leprae Br4923
Mycobacterium leprae TN
Mycobacterium tuberculosis H37Ra
Mycobacterium tuberculosis H37RvAE
Mycobacterium tuberculosis H37RvCO
Mycobacterium tuberculosis H37RvHA
Mycobacterium tuberculosis H37RvJO
Neisseria gonorrhoeae 1291

Neisseria gonorrhoeae DGI2
Neisseria gonorrhoeae FA 1090
Neisseria gonorrhoeae PID18
Neisseria gonorrhoeae SK-93-1035
Neisseria meningitidis 2003022
Neisseria meningitidis 2004032
Neisseria meningitidis 64182
Neisseria meningitidis 93003
Neisseria meningitidis NM3222
Staphylococcus aureus subsp aureus ATCC 51811
Staphylococcus aureus subsp aureus ATCC BAA-39
Staphylococcus aureus subsp aureus COL
Staphylococcus aureus subsp aureus MRSA131
Staphylococcus aureus subsp aureus USA300 TCH1516
Staphylococcus epidermidis NIH051668
Staphylococcus epidermidis VCU120
Staphylococcus epidermidis VCU139
Staphylococcus epidermidis W23144
Staphylococcus epidermidis WI05
Streptococcus agalactiae A909
Streptococcus agalactiae CCUG 24810
Streptococcus agalactiae GB00247
Streptococcus agalactiae GB00951
Streptococcus agalactiae ILRI005
Streptococcus dysgalactiae subsp dysgalactiae ATCC 27957
Streptococcus dysgalactiae subsp equisimilis 167
Streptococcus dysgalactiae subsp equisimilis ATCC 12394
Streptococcus dysgalactiae subsp equisimilis GGS 124
Streptococcus dysgalactiae subsp equisimilis RE378
Streptococcus mutans TCI-110
Streptococcus mutans TCI-149
Streptococcus mutans TCI-223
Streptococcus mutans TCI-400
Streptococcus mutans TCI-92
Streptococcus pneumoniae ATCC 700669
Streptococcus pneumoniae Hungary19A-6
Streptococcus pneumoniae SP195
Streptococcus pneumoniae Taiwan19F-14
Streptococcus pneumoniae TCH843119A
Streptococcus pyogenes GA03805
Streptococcus pyogenes GA16797
Streptococcus pyogenes GA19681
Streptococcus pyogenes MGAS2096
Streptococcus pyogenes UTMEM-1
Ureaplasma urealyticum 2608

Ureaplasma urealyticum serovar 11 str ATCC 33695
Ureaplasma urealyticum serovar 2 str ATCC 27814
Ureaplasma urealyticum serovar 7 str ATCC 27819
Ureaplasma urealyticum serovar 9 str ATCC 33175
**GI microbiome**
Anaerobaculum hydrogeniformans ATCC BAA-1850
Anaerostipes caccae DSM 14662
Anaerostipes sp. 3 2 56FAA
Bacteroides cellulosilyticus DSM 14838
Bacteroides clarus YIT 12056
Bacteroides eggerthii DSM 20697
Bacteroides sp. 3 1 19
Bacteroides sp. D22
Bacteroides xylanisolvens SD CC 1b
Bacteroides xylanisolvens SD CC 2a
Bifidobacterium breve DSM 20213 = JCM 1192
Bifidobacterium sp. 12 1 47BFAA
Citrobacter youngae ATCC 29220
Clostridiales butyrate-prod
Clostridiales butyrate-producing bacter
Clostridiales butyrate-producing bacterium SS
Clostridiales butyrate-producing bacterium SSC/2
Clostridium sp. M62/1
Clostridium sp. SS2/1
Coprobacillus sp. 8 2 54BFAA
Coprococcus sp. HPP0074
Corynebacterium sp. HFH0082
Edwardsiella tarda ATCC 23685
Enterobacter cancerogenus ATCC 35316
Enterococcus faecalis TX2134
Erysipelotrichaceae bacterium 21 3
Escherichia coli 4 1 47FAA
Escherichia coli MS 60-1
Escherichia coli MS 69-1
Escherichia coli MS 78-1
Escherichia coli MS 84-1
Eubacterium rectale M104/1
Faecalibacterium prausnitzii M21/2
Fusobacterium mortiferum ATCC 9817
Fusobacterium varium ATCC 27725
Hafnia alvei ATCC 51873
Helicobacter pylori GAM101Biv
Helicobacter pylori GAM246Ai
Helicobacter pylori GAM252T

Helicobacter pylori GAM83Bi
Helicobacter pylori GAM93Bi
Helicobacter pylori GAM96Ai
Helicobacter pylori HP116Bi
Helicobacter pylori HP250BFiii
Helicobacter pylori HP250BSi
Klebsiella pneumoniae subsp. pneumoniae WGLW3
Lachnospiraceae bacterium 2 1 58FAA
Lachnospiraceae bacterium 3 1 57FAA CT1
Lachnospiraceae bacterium 5 1 57FAA
Lachnospiraceae bacterium 5 1 63FAA
Lachnospiraceae bacterium 7 1 58FAA
Lactobacillus reuteri MM4-1A
Lactobacillus reuteri SD2112
Listeria innocua ATCC 33091
Megamonas hypermegale ART12/1
Methanobrevibacter smithii DSM 2375
Paraprevotella xylaniphila YIT 11841
Phascolarctobacterium succinatutens YIT 12067
Prevotella oralis HGA0225
Roseburia intestinalis M50/1
Roseburia intestinalis XB6B4
Ruminococcus obeum A2-162
Ruminococcus torques L2-14
Succinatimonas hippei YIT 12066
Sutterella wadsworthensis 2 1 59BFAA
Veillonella sp. 6 1 27
Weissella paramesenteroides ATCC 33313

Supplemental Table 2. Composition of TCEM I Proteome Datasets.

TCEM Count	Human	Pathogen	Microbiome	Percent of 3.2 million
2,059,860	1	1	1	64.37%
218,638	1	0	1	6.83%
423,041	0	1	1	13.22%
204,804	0	0	1	6.40%
49,248	1	1	0	1.54%
62,238	1	0	0	1.94%
53,434	0	1	0	1.67%
128,737	0	0	0	4.02%

Supplemental Table 3. Fractional composition of Proteomic Datasets and Fractional Matches of IgV TCEM IIa motifs.

Group	Count	Percent of 3.2 million	IGV coverage
Human	2,389,984	74.7%	12.0%
Human excluded	810,016	25.3%	5.3%
Pathogen	2,585,583	80.1%	11.9%
Pathogen excluded	614,417	19.2%	3.6%
Microbiome	2,906,343	90.8%	11.0%
Microbiome excluded	293,657	9.2%	2.9%
Not Found in Human, Pathogen or Microbiome	128,737	4.0%	2.1%

Supplemental Table 4. Shannon Entropy of TCEM I motifs in Different Proteome Subsets.

Variable	by Variable	Spearman ρ	Prob>|ρ|
log2 N Human freq	log2 N Microbiome freq	0.5125	<.0001*
log2N Pathogen freq	log2 N Microbiome freq	0.7347	<.0001*
log2N Pathogen freq	log2 N Human freq	0.4933	<.0001*
log2 IGLV freq	log2 N Microbiome freq	0.0585	<.0001*
log2 IGLV freq	log2 N Human freq	0.0764	<.0001*
log2 IGLV freq	log2N Pathogen freq	0.0543	<.0001*
log2 IGHV freq	log2 N Microbiome freq	0.0711	<.0001*
log2 IGHV freq	log2 N Human freq	0.0621	<.0001*
log2 IGHV freq	log2N Pathogen freq	0.0560	<.0001*
log2 IGHV freq	log2 IGLV freq	0.2933	<.0001*

Supplemental Table 5. Rank Correlation of TCEM I Occurrence between Different Proteomic Subsets.

I

Source	Shannon Entropy
Sum (Human and Pathogen)	20.31
Sum (Human and Microbiome)	20.59
Sum (Human)	20.35
Sum (IGVL)	12.22
Sum (IGHV)	12.37

Supplemental Table 6. TCEM IIa content of Pathogen proteomes.
The mean represents the percentage of motifs of all proteins in the entire proteome that match a TCEM IIa in the IgV database. ANOVA comparisons for all pairs using Tukey-

Kramer HSD. Organisms not connected by the same letter are significantly different (α = 0.05).

**Organism**															**Mean**
Mycobacterium tuberculosis	A														22.84
Mycobacterium bovis	A														22.80
Mycobacterium abscessus		B													22.16
Burkholderia pseudomallei		B													22.03
Burkholderia mallei			C												21.74
Burkholderia cepacia				D											21.39
Burkholderia cenocepacia				D											21.38
Mycobacterium leprae				D											21.34
Bordetella bronchiseptica					E										20.99
Bordetella pertussis					E										20.98
Brucella melitensis						F									19.46
Neisseria gonorrhoeae							G								17.92
Neisseria meningitidis								H							17.66
Chlamydia trachomatis								H							17.47
Streptococcus mutans									I						16.80
Streptococcus dysgalactiae									I						16.77
Streptococcus pyogenes									I	J					16.66
Streptococcus agalactiae										J	K				16.45
Streptococcus pneumoniae											K				16.39
Coxiella burnetii											K	L			16.21
Francisella tularensis												L			16.16
Francisella novicida												L			16.04
Staphylococcus aureus													M		15.39
Clostridium difficile													M		15.32
Clostridium perfringens													M		15.22
Staphylococcus epidermidis													M		15.21
Ureaplasma urealyticum														N	13.94

Levels not connected by same letter are significantly different.

Supplemental Table 7. TCEM IIa content of Microbiome proteomes.
The mean represents the percentage of motifs of all proteins in the entire proteome that match a TCEM IIa in the IgV database. ANOVA comparisons for all pairs using Tukey-Kramer HSD. Organisms not connected by the same letter are significantly different ($\alpha = 0.05$).

**Organism**																	**Mean**
Corynebacterium	A																20.81
Bifidobacterium		B															19.35
Sutterella		B															19.09
Edwardsiella			C														18.56
Klebsiella			C														18.49
Enterobacter				D													18.03
Citrobacter				D													17.88
Escherichia					E												17.57
Hafnia					E	F											17.51
Faecalibacterium					E	F	G										17.28
Anaerobaculum					E	F	G										17.26
Weissella					E	F	G										17.18
Veillonella						F	G	H									17.09
Phascolarctobacterium							G	H	I								16.87
Succinatimonas								H	I	J							16.63
Lachnospiraceae									I								16.56
Prevotella									I	J	K						16.46
Paraprevotella									I	J	K						16.43
Bacteroides										J	K						16.37
Anaerostipes										J	K	L					16.27
Listeria										J	K	L	M				16.22
Clostridium											K	L	M				16.21
Eubacterium											K	L	M				16.10
Roseburia													M				15.96
Lactobacillus												L	M	N			15.91
Ruminococcus													M				15.91
Enterococcus													M	N			15.89
Methanobrevibacter												L	M	N			15.87
Megamonas													M	N	O		15.78
Clostridiales														N	O	P	15.45
Erysipelotrichaceae															O	P	15.33
Coprococcus																P	15.27
Helicobacter																P	15.17
Fusobacterium																P	15.12
Coprobacillus																P	15.12

Levels not connected by same letter are significantly different.

Supplemental Table 8. TCEM I content of Pathogen proteomes.
The mean represents the percentage of motifs of all proteins in the entire proteome that match a TCEM IIa in the IgV database. ANOVA comparisons for all pairs using Tukey-Kramer HSD. Organisms not connected by the same letter are significantly different (α = 0.05).

**Organism**															**Mean**
Mycobacterium tuberculosis	A														24.22
Mycobacterium bovis	A														24.14
Mycobacterium abscessus		B													23.29
Burkholderia pseudomallei			C												22.91
Burkholderia mallei				D											22.62
Mycobacterium leprae				D	E										22.45
Burkholderia cepacia					E										22.25
Burkholderia cenocepacia					E										22.22
Bordetella bronchiseptica						F									21.88
Bordetella pertussis						F									21.85
Brucella melitensis							G								20.41
Neisseria gonorrhoeae								H							18.78
Chlamydia trachomatis								H	I						18.65
Neisseria meningitidis									I						18.44
Streptococcus mutans										J					17.67
Streptococcus pyogenes										J	K				17.45
Streptococcus dysgalactiae										J	K				17.41
Streptococcus agalactiae										J	K				17.40
Francisella tularensis											K	L			17.23
Streptococcus pneumoniae												L			17.11
Coxiella burnetii												L			17.11
Francisella novicida												L			17.01
Staphylococcus aureus													M		16.29
Clostridium difficile													M		16.23
Staphylococcus epidermidis													M		16.11
Clostridium perfringens													M		16.06
Ureaplasma urealyticum														N	14.93

Levels not connected by same letter are significantly different.

Supplemental Table 9. TCEM I content of Microbiome proteomes.
The mean represents the percentage of motifs of all proteins in the entire proteome that match a TCEM IIa in the IgV database. ANOVA comparisons for all pairs using Tukey-Kramer HSD. Organisms not connected by the same letter are significantly different ($\alpha = 0.05$).

**Organism**																				**Mean**
Corynebacterium	A																			21.98
Bifidobacterium		B																		20.28
Sutterella		B																		19.97
Edwardsiella			C																	19.43
Klebsiella			C																	19.34
Enterobacter				D																18.90
Citrobacter				D	E															18.72
Escherichia					E															18.48
Hafnia					E	F														18.39
Anaerobaculum						F	G													18.09
Faecalibacterium						F	G	H												17.98
Weissella						F	G	H												17.96
Veillonella							G	H	I											17.74
Phascolarctobacterium							G	H	I	J	K									17.56
Succinatimonas								H	I	J	K	L								17.50
Lachnospiraceae									I	J										17.38
Prevotella									I	J	K	L	M							17.31
Paraprevotella									I	J	K	L	M							17.29
Bacteroides										J	K	L								17.22
Clostridium											K	L	M							17.11
Anaerostipes												L	M	N						17.06
Listeria												L	M	N		P				16.99
Methanobrevibacter													M	N	O	P				16.74
Enterococcus														N	O	P				16.65
Roseburia															O		Q			16.57
Ruminococcus															O		Q			16.56
Eubacterium															O	P	Q	R		16.54
Lactobacillus															O	P	Q	R		16.49
Megamonas															O		Q	R	S	16.23
Clostridiales																	Q	R	S	16.15
Coprococcus																		R	S	16.07
Erysipelotrichaceae																			S	16.01
Fusobacterium																			S	15.99
Coprobacillus																			S	15.99
Helicobacter																			S	15.85

Levels not connected by same letter are significantly different.

Supplemental Table 10: Source proteins used in the Figure 12 comparison.
The human proteins are identified by their UniProt accession number and the *Burkholderia pseudomallei* by the PATRIC accession number.

	A6NIH7 U119B_HUMAN Protein unc-119 homolog B OS_*Homo sapiens* GN_UNC119B PE_1 SV_1
	E7EST6 E7EST6_HUMAN Plakophilin-4 OS_ *Homo sapiens* GN_PKP4 PE_1 SV_1
	H0YFB1 H0YFB1_HUMAN Plakophilin-4 (Fragment) OS_ *Homo sapiens* GN_PKP4 PE_1 SV_1
	O60423 AT8B3_HUMAN Phospholipid-transporting ATPase IK OS_ *Homo sapiens* GN_ATP8B3 PE_2 SV_4
a	Q6UXG8 BTNL9_HUMAN Butyrophilin-like protein 9 OS_ *Homo sapiens* GN_BTNL9 PE_2 SV_1
	Q8N414 PGBD5_HUMAN PiggyBac transposable element-derived protein 5 OS_ *Homo sapiens* GN_PGBD5 PE_1 SV_3
	Q8NFY9 KBTB8_HUMAN Kelch repeat and BTB domain-containing protein 8 OS_ *Homo sapiens* GN_KBTBD8 PE_2 SV_2
	Q96DT5 DYH11_HUMAN Dynein heavy chain 11
	Q99569 PKP4_HUMAN Plakophilin-4 OS_ *Homo sapiens* GN_PKP4 PE_1 SV_2
	Q9HCM3 K1549_HUMAN UPF0606 protein KIAA1549 OS *Homo sapiens* GN_KIAA1549 PE_1 SV_4
b	19271492 : Long-chain-fatty-acid--CoA ligase (EC 6_2_1_3) *Burkholderia pseudomallei* K96243
	19271494 : Long-chain-fatty-acid--CoA ligase (EC 6_2_1_3) *Burkholderia pseudomallei* K96243
	19271496 : Long-chain-fatty-acid--CoA ligase (EC 6_2_1_3) *Burkholderia pseudomallei* K96243

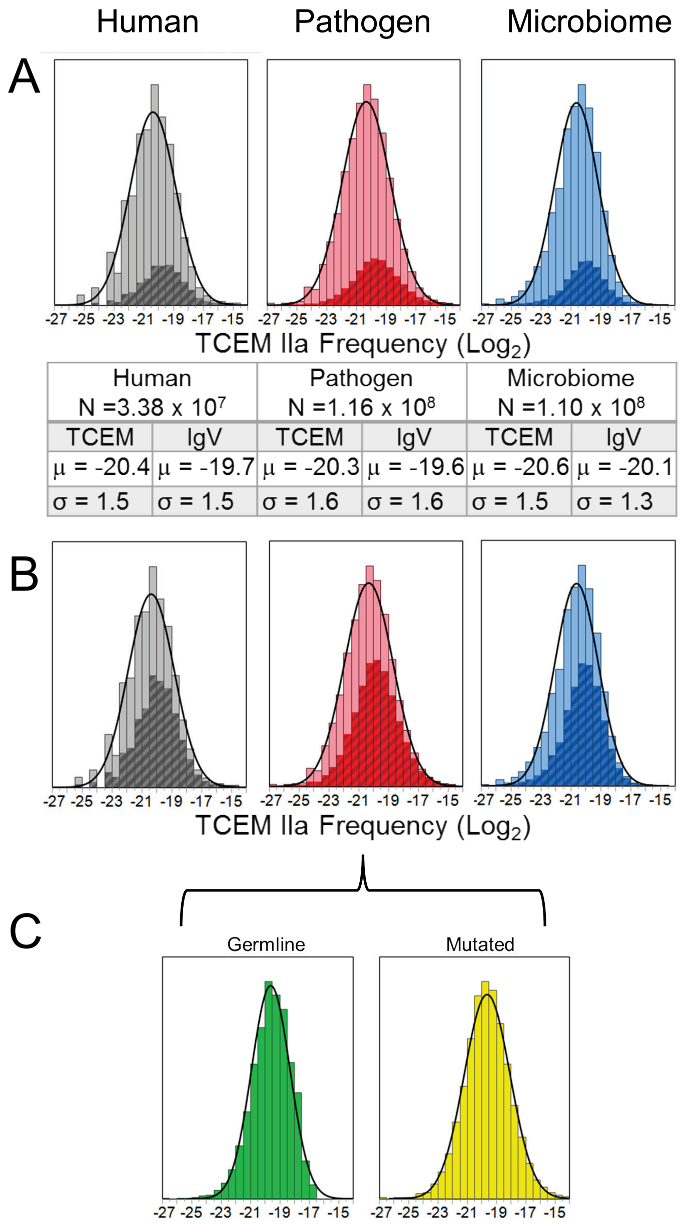

Human N =3.38 x 10^7		Pathogen N =1.16 x 10^8		Microbiome N =1.10 x 10^8	
TCEM	IgV	TCEM	IgV	TCEM	IgV
μ = -20.4	μ = -19.7	μ = -20.3	μ = -19.6	μ = -20.6	μ = -20.1
σ = 1.5	σ = 1.5	σ = 1.6	σ = 1.6	σ = 1.5	σ = 1.3

Supplemental Figure 1. Histograms of TCEM I in each proteome set analyzed. (A) The frequency of occurrence of each of the possible 20^5 TCEM I pentamers was computed for all of the proteins in each of the proteomes. The fit of a frequency-weighted normal distribution to each of the datasets is shown by the solid curve. The number of motifs and the parameters of the normal distribution curve are given in the inset. The cross hatched region within the histogram corresponds to the frequency of IgV-origin TCEM I. X axis shows the log2 of frequency of occurrence.

(B) The background frequency distribution is as in (A) but showing the overlap of IgV TCEM I pentamers when with a single conservative amino acid replacement is permitted.

(C) Separation of the IgV origin pentamers for the Pathogen proteomes showing germline-origin and somatic hypermutated-origin subset distributions of IgV TCEM I.

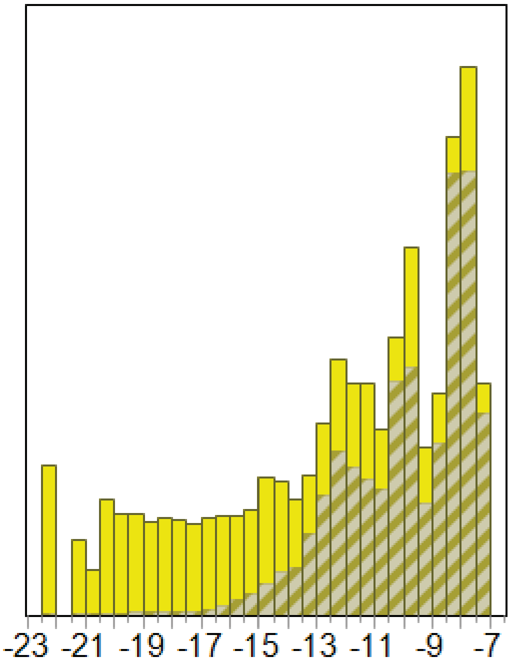

Supplemental Figure 2 Frequency distributions of IgV-origin TCEM I motifs for the Pathogen proteome

Shows the frequency distribution of the IgV TCEM I motifs in the Pathogen proteome set as shown by the cross hatched insets in Figure 2A. The bars in the histogram represent the fraction of the total motifs at the particular frequency. The crosshatched region depicts the fraction of the total at the particular frequency that is of germline origin and the solid portion of the bar is attributable to somatic hypermutation origin motifs. X axis shows the log2 of frequency of occurrence.

Supplemental Figure 3. Frequency distributions of the proteomic TCEM IIa subsets which overlap with IgV set.
Shows the frequency distribution histogram of motifs that match the indicated IgV frequency classification (FC). The FC classification scheme is a log2-based system for categorizing the frequency of occurrence of a particular motif in IgV (See Bremel and Homan, 2014 Front Immunol). For example, an FC3 = $1/2^3$ = 12.5% of all B cell clonotypes and comprises a subset of 137 motifs. Multiple copies of those 137 motifs are found in each of the proteomes (Pathogen average =109). Likewise, FC5 = $1/2^5$ = 1/32 or about 3% of all clonotypes and comprises a subset of 507 motifs and are found an average of 322 times in each of the Pathogen proteomes. As in the IgV the motifs are found in the proteomes with many different groove exposed motifs (GEM) which will confer different binding affinities to the pMHC. A skewing is seen in the histograms for FC1 to FC7 favoring a higher frequency match. This is also evidenced by approximately one log2 unit or a doubling in the mean as compared to the frequency distributions for the entire datasets shown in Figure 1 (i.e. a mean shift from approximately -20.5 to -19.5). Thus, it appears that the somatic mutation process tends to be biased towards producing motifs that are commonly found in other proteins.

TCEM IIA

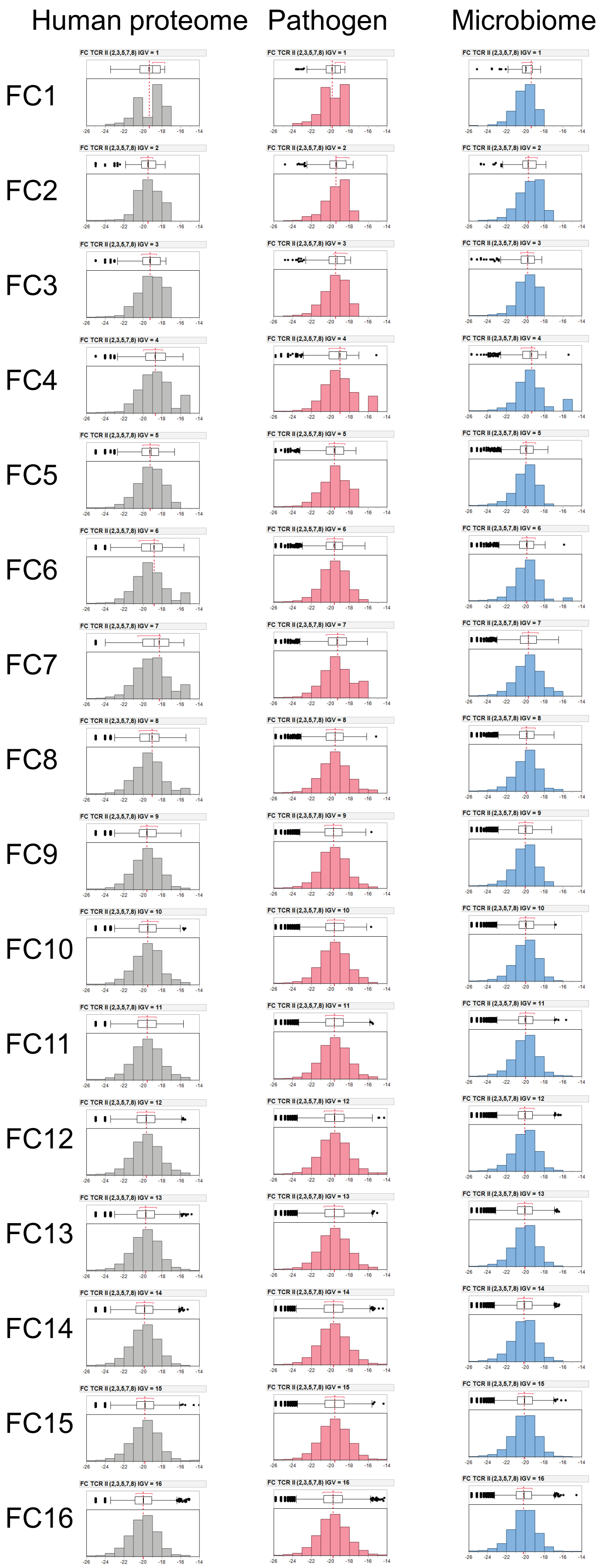

Supplemental Figure 4. Frequency distributions of the proteomic TCEM I subsets which overlap with IgV set.
Shows the frequency distribution histogram of motifs that match the indicated IgV frequency classification (FC). See legend of Supplemental Figure 3 for more detailed explanation.

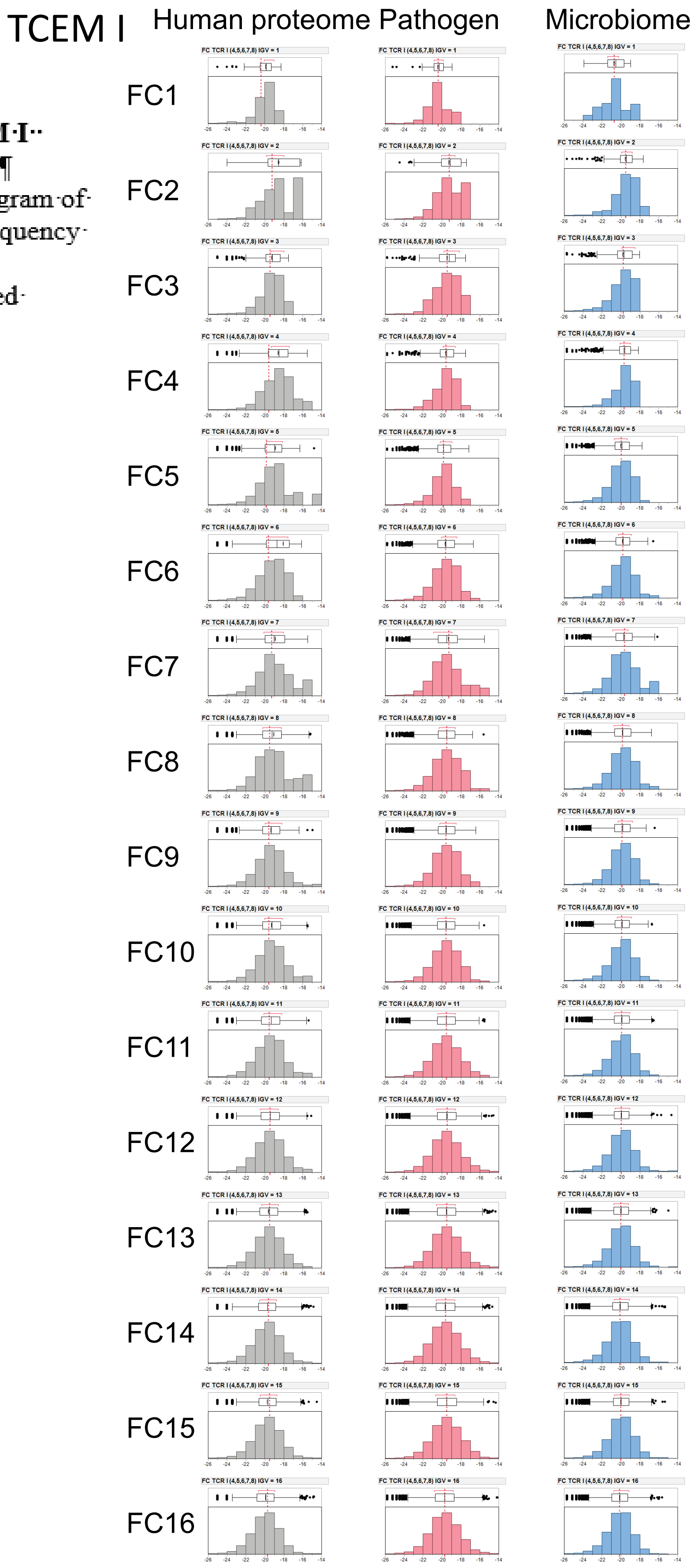

TCEM I

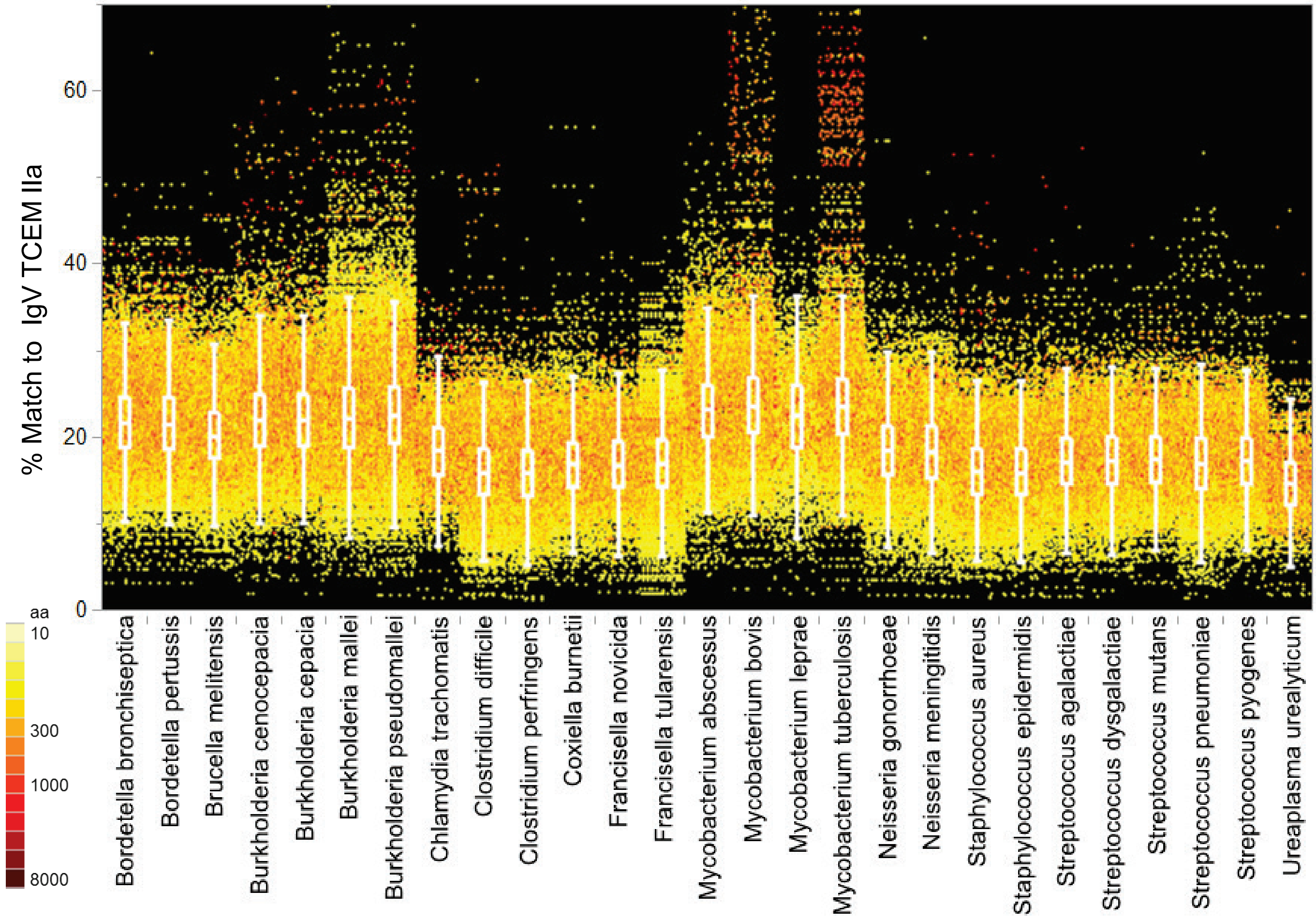

Supplemental Figure 5. IgV TCEM I frequency patterns within the Pathogen proteomes.

X axis: Groups of five Pathogen total proteomes are clustered by genus with each protein represented by a colored dot. Y axis shows the percentage match of IgV TCEM I in each protein. Each dot represents the metric computed for a single protein in the proteome of the different organisms. The color of the dot is proportional to the molecular weight of the protein. The inset box shows the interquartile range of the metric in each of the proteomes and the whiskers indicate the 10% and 90% of the metric. For the human proteome the average IgV content is 17.4%.

TCEM I

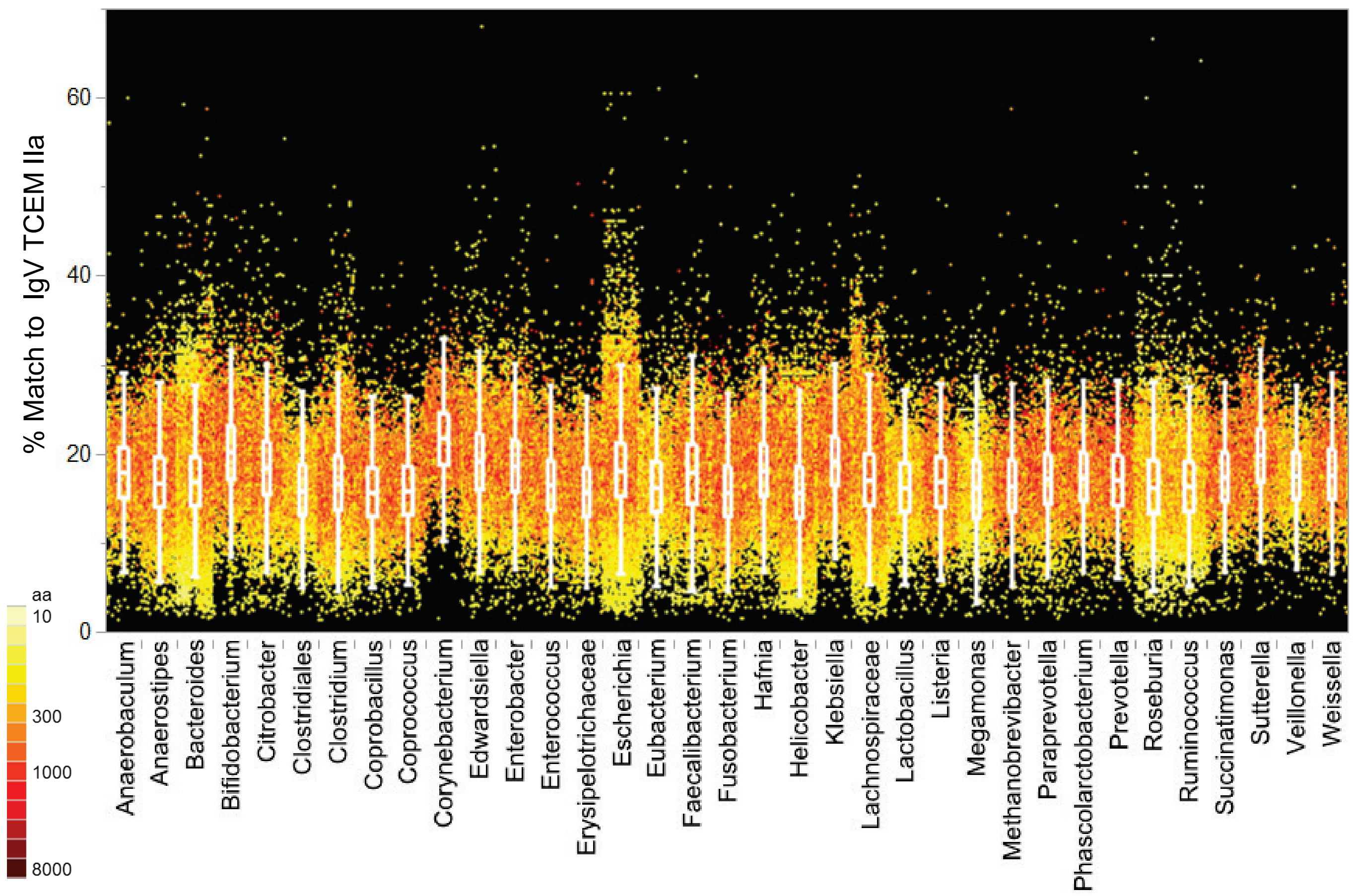

Supplemental Figure 6. IgV TCEM I frequency patterns in the Microbiome proteomes.
X axis: Groups of Microbiome proteomes are clustered by genus with each protein represented by a colored dot. Y axis shows the percentage match of IgV TCEM I in each protein. Each dot represents the metric computed for a single protein in the proteome of the different organisms. The color of the dot is proportional to the molecular weight of the protein. The inset box shows the interquartile range of the metric in each of the proteomes and the whiskers indicate the 10% and 90% of the metric.

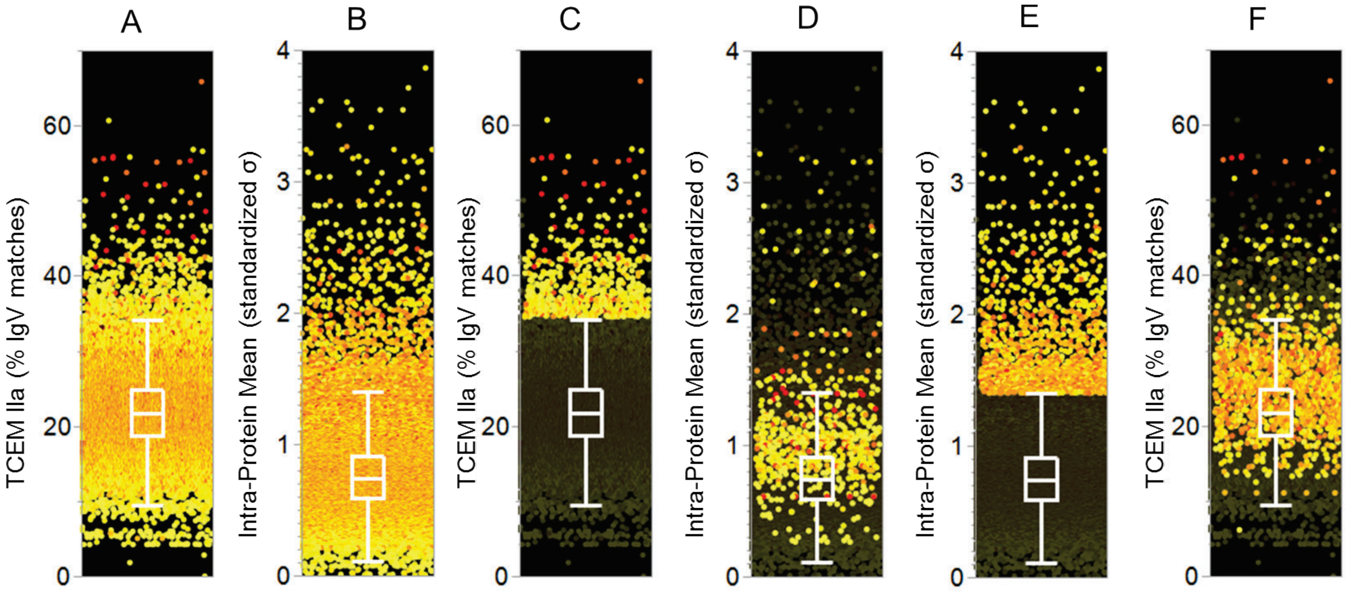

Supplemental Figure 7: Comparison of IgV and human proteome TCEM IIa frequency patterns for *Burkholderia pseudomallei*.

B. pseudomallei proteomes shown in Figure 5 are examined: (A) Percent match to IvG TCEM IIa as in Figure 5; (B) Human proteome TCEM frequency usage applied to these proteins; (C) Outliers from (A) highlighted; (D) Human proteome TCEM frequency usage applied to outliers in (C); in (E) and (F) the reverse comparison shows IgV TCEM frequency usage of the Human proteome frequency outliers from (B). Outliers are defined as being outside the area defined by the whiskers of the box plot (1.5 x the interquartile range shown by the box).

Supplemental Figure 8. Tree-map of TCEM I frequency patterns in the Pathogen proteomes. Each organism shows a different pattern of use of the possible 20^5 total motifs. This is a binary absence/presence depiction analogous to a Venn diagram. It comprises approximately 1.5 million different patterns of 110 million motifs. To construct this map tallies were made of each of the TCEM I motifs found in each organism's proteome. A binary coding scheme was used to score the presence or absence of a particular motif (of 20^5 total) in a proteome. The size of the rectangle is proportional to the number of motifs. The lightly colored rectangles indicate relatively rare motifs and the dark rectangles indicate common motifs. The large rectangles indicate motifs unique to one or a few species; the organism corresponding to each large rectangle is shown in the accompanying tabulation along with the number of motifs in each of the N proteome and the fraction of the total of each proteome of the organism represented by those motifs.

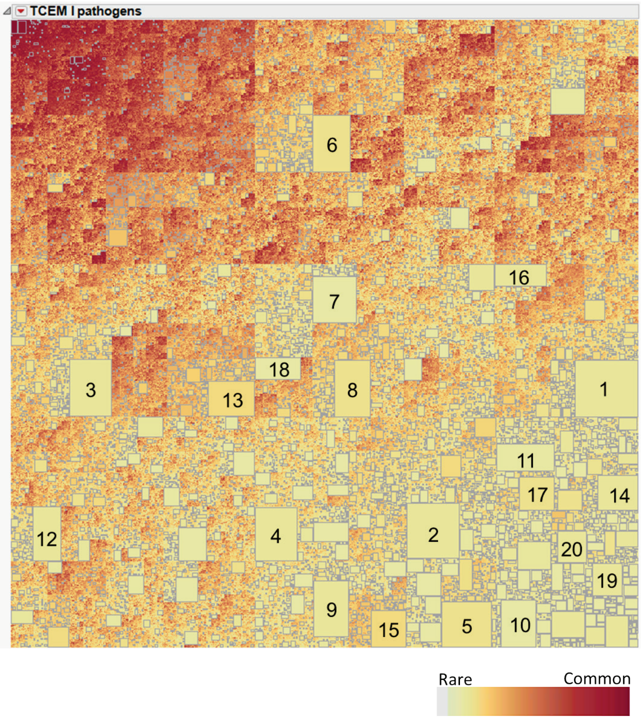

Box	Genera	N	%	Unique Motifs
1	*Clostridium difficile*	5	4.25%	23,522
2	*Mycobacterium abscessus*	5	3.25%	19,787
3	*Brucella melitensis*	5	3.22%	15,766
4	*Clostridium perfringens*	5	3.17%	15,347
5	*Mycobacterium bovis*	5	2.89%	15,069
	Mycobacterium tuberculosis	5	2.83%	
6	*Bordetella bronchiseptica*	5	2.63%	14,875
	Bordetella pertussis	5	2.95%	
7	*Burkholderia cepacia*	1	1.79%	13,924
8	*Burkholderia mallei*	5	2.27%	13,627
8	*Burkholderia pseudomallei*	5	1.95%	
9	*Coxiella burnetii*	5	3.59%	13,244
10	*Staphylococcus epidermidis*	1	2.55%	10,947
11	*Mycobacterium leprae*	2	2.87%	10,823
12	*Burkholderia cenocepacia*	3	1.50%	10,420
	Burkholderia cepacia	2	1.38%	
13	*Burkholderia cenocepacia*	3	1.49%	10,371
	Burkholderia cepacia	2	1.38%	
	Burkholderia mallei	5	1.73%	
	Burkholderia pseudomallei	5	1.48%	
14	*Staphylococcus aureus*	5	1.93%	9,012
15	*Francisella novicida*	5	2.27%	8,234
	Francisella tularensis	10	2.42%	
16	*Chlamydia trachomatis*	5	3.68%	8,122
17	*Neisseria gonorrhoeae*	5	2.07%	7,622
	Neisseria meningitidis	5	2.05%	
18	*Burkholderia cepacia*	1	0.99%	7,174
19	*Streptococcus pneumoniae*	5	1.70%	6,596
20	*Streptococcus agalactiae*	5	1.56%	6,198

Supplemental Figure 9. Tree map of combined TCEM I frequency patterns in the Pathogen proteomes and the Human proteome.
The algorithm is the same as used in Figure 7 but in this case the human proteome was also included in the analysis. This shows the inter-relationship between the motifs of the Pathogen and the Human. The accompanying tabulation of the 20 largest boxes indicates the composition depicted in these rectangles.

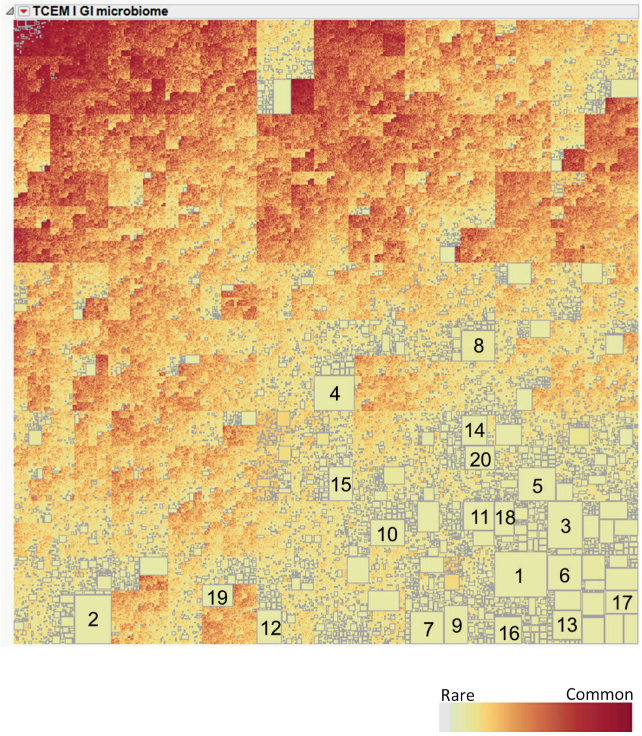

Box	Organism	%	Unique motifs
1	*Lachnospiraceae bacterium* 3_1_57FAA_CT1	1.75%	18,241
2	*Bacteroides cellulosilyticus* DSM 14838	1.30%	13,295
3	*Lachnospiraceae bacterium* 7_1_58FAA	1.54%	12,567
4	*Bacteroides sp.* 3_1_19	1.24%	10,616
5	*Erysipelotrichaceae bacterium* 21_3	1.30%	9,414
6	*Paraprevotella xylaniphila* YIT 11841	1.29%	9,298
7	*Hafnia alvei* ATCC 51873	1.02%	7,978
8	*Bacteroides sp.* D22	0.79%	7,591
8	*Bacteroides xylanisolvens* SD CC 1b	0.80%	7,591
8	*Bacteroides xylanisolvens* SD CC 2a	0.80%	7,591
9	*Klebsiella pneumoniae subsp. pneumoniae* WGLW3	0.85%	6,986
10	*Coprobacillus sp.* 8_2_54BFAA	1.04%	6,803
11	*Faecalibacterium prausnitzii* M21/2	1.16%	6,549
12	*Clostridium sp.* M62/1	0.93%	6,298
13	*Prevotella oralis* HGA0225	1.10%	6,240
14	*Edwardsiella tarda* ATCC 23685	0.98%	5,958
15	*Citrobacter youngae* ATCC 29220	0.72%	5,902
16	*Lachnospiraceae bacterium* 5_1_57FAA	0.87%	5,401
17	*Sutterella wadsworthensis* 2_1_59BFAA	1.18%	5,383
18	*Lachnospiraceae bacterium* 2_1_58FAA	0.84%	5,371
19	*Bacteroides eggerthii* DSM 20697	0.70%	5,350
20	*Enterobacter cancerogenus* ATCC 35316	0.69%	5,262

Supplemental Figure 10. Tree map of TCEM IIa frequency patterns in the Microbiome proteomes. See Figure 8 for more complete description.

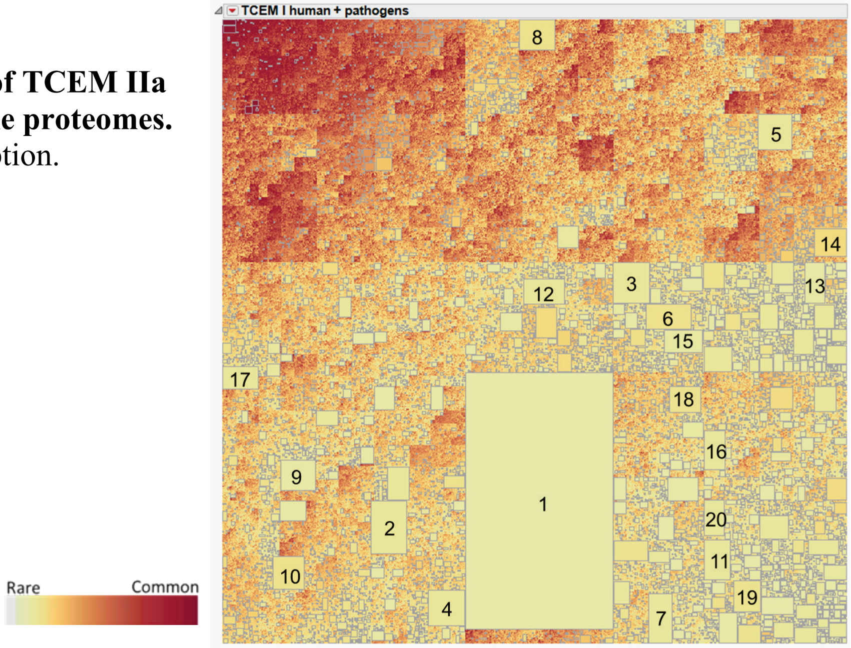

Box	Organism	Unique motifs
1	Human	275,518
2	Human / Clostridium difficile ATCC 43255 / Clostridium difficile ATCC 9689 / Clostridium difficile CD165 / Clostridium difficile DA00212 / Clostridium difficile Y270	14,189
3	Human / Mycobacterium abscessus 4S-0303 / Mycobacterium abscessus 5S-1212 / Mycobacterium abscessus 6G-0212 / Mycobacterium abscessus M156 / Mycobacterium abscessus V06705	11,714
4	Human / Brucella melitensis 043 / Brucella melitensis ATCC 23457 / Brucella melitensis B115 / Brucella melitensis M5 / Brucella melitensis S66	9,244
5	Human / Clostridium perfringens ATCC 13124 / Clostridium perfringens CPE str F4969 / Clostridium perfringens E str JGS1987 / Clostridium perfringens JJC / Clostridium perfringens str 13	9,145
6	Human / Mycobacterium bovis BCG str ATCC 35733 / Mycobacterium bovis BCG str ATCC 35740 / Mycobacterium bovis BCG str ATCC 35743 / Mycobacterium bovis BCG str Glaxo / Mycobacterium bovis BCG str Pasteur 1173P2 / Mycobacterium tuberculosis H37Ra / Mycobacterium tuberculosis H37RvAE / Mycobacterium tuberculosis H37RvCO / Mycobacterium tuberculosis H37RvHA / Mycobacterium tuberculosis H37RvJO	8,896
7	Human / Bordetella bronchiseptica 2371640 / Bordetella bronchiseptica 253 / Bordetella bronchiseptica D445 / Bordetella bronchiseptica RB50 / Bordetella bronchiseptica SEAT0006 / Bordetella pertussis B1920 / Bordetella pertussis Bp H897 / Bordetella pertussis Bp SEAT 0004 / Bordetella pertussis CHLA15 / Bordetella pertussis Tohama I	8,814
8	Human / Burkholderia mallei ATCC 10399 / Burkholderia mallei ATCC 23344 / Burkholderia mallei FMH / Burkholderia mallei JHU / Burkholderia mallei NCTC 10247 / Burkholderia pseudomallei 1026b / Burkholderia pseudomallei 1106a / Burkholderia pseudomallei 1106b / Burkholderia pseudomallei K96243 / Burkholderia pseudomallei MSHR305	8,726
9	Clostridium difficile ATCC 43255 / Clostridium difficile ATCC 9689 / Clostridium difficile CD165 / Clostridium difficile DA00212 / Clostridium difficile Y270	8,654
10	Human / Burkholderia cepacia ATCC 25416	8,253
11	Mycobacterium abscessus 4S-0303 / Mycobacterium abscessus 5S-1212 / Mycobacterium abscessus 6G-0212 / Mycobacterium abscessus M156 / Mycobacterium abscessus V06705	7,853
12	Human / Coxiella burnetii Cb185 / Coxiella burnetii Dugway 5J108-111 / Coxiella burnetii RSA 331 / Coxiella burnetii RSA 493 / Coxiella burnetii Z3055	7,792
13	Human / Burkholderia cenocepacia AU 1054 / Burkholderia cenocepacia H111 / Burkholderia cenocepacia KC-01 / Burkholderia cepacia ATCC 25416 / Burkholderia cepacia Bu72	6,438
14	Human / Staphylococcus epidermidis VCU139	6,407
15	Human / Burkholderia cenocepacia AU 1054 / Burkholderia cenocepacia H111 / Burkholderia cenocepacia KC-01 / Burkholderia cepacia ATCC 25416 / Burkholderia cepacia Bu72 / Burkholderia mallei ATCC 10399 / Burkholderia mallei ATCC 23344 / Burkholderia mallei FMH / Burkholderia mallei JHU / Burkholderia mallei NCTC 10247 / Burkholderia pseudomallei 1026b / Burkholderia pseudomallei 1106a / Burkholderia pseudomallei 1106b / Burkholderia pseudomallei K96243 / Burkholderia pseudomallei MSHR305	6,397
16	Human / Mycobacterium leprae Br4923 / Mycobacterium leprae TN	6,377
17	Brucella melitensis 043 / Brucella melitensis ATCC 23457 / Brucella melitensis B115 / Brucella melitensis M5 / Brucella melitensis S66	6,216
18	Bordetella bronchiseptica 2371640 / Bordetella bronchiseptica 253 / Bordetella bronchiseptica D445 / Bordetella bronchiseptica RB50 / Bordetella bronchiseptica SEAT0006 / Bordetella pertussis B1920 / Bordetella pertussis Bp H897 / Bordetella pertussis Bp SEAT 0004 / Bordetella pertussis CHLA15 / Bordetella pertussis Tohama I	6,068
19	Mycobacterium bovis BCG str ATCC 35733 / Mycobacterium bovis BCG str ATCC 35740 / Mycobacterium bovis BCG str ATCC 35743 / Mycobacterium bovis BCG str Glaxo / Mycobacterium bovis BCG str Pasteur 1173P2 / Mycobacterium tuberculosis H37Ra / Mycobacterium tuberculosis H37RvAE / Mycobacterium tuberculosis H37RvCO / Mycobacterium tuberculosis H37RvHA / Mycobacterium tuberculosis H37RvJO	5,935
20	Burkholderia cepacia ATCC 25416	5,673

Supplemental Figure 11. Intersection between the Human proteome and the Microbiome. See Figure 10 for more complete description.

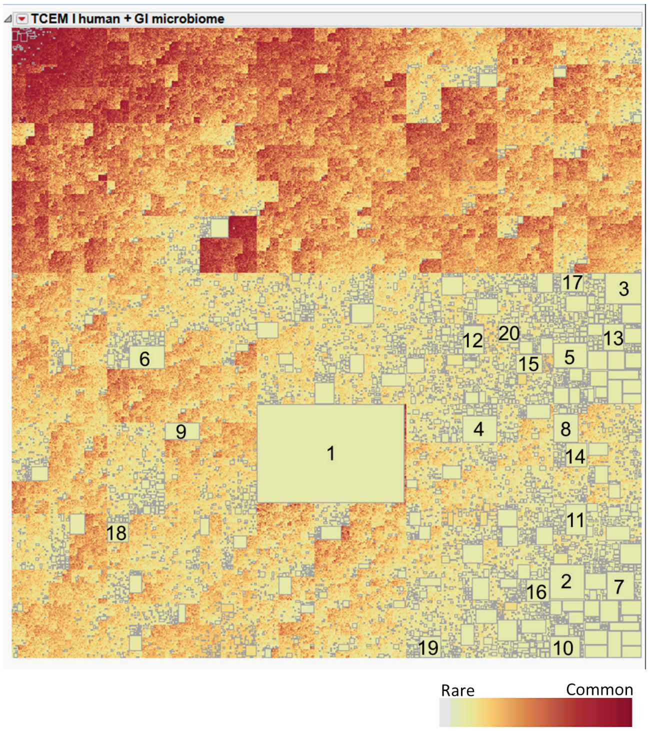

Box	Organism	Unique motifs
1	Human	111,486
2	Lachnospiraceae bacterium 3_1_57FAA_CT1	9,496
3	Human / Lachnospiraceae bacterium 3_1_57FAA_CT1	8,745
4	Bacteroides cellulosilyticus DSM 14838	7,072
5	Human / Lachnospiraceae bacterium 7_1_58FAA	6,547
6	Human / Bacteroides cellulosilyticus DSM 14838	6,223
7	Lachnospiraceae bacterium 7_1_58FAA	6,020
8	Bacteroides sp. 3_1_19	5,646
9	Human / Bacteroides sp. 3_1_19	4,970
10	Paraprevotella xylaniphila YIT 11841	4,932
11	Erysipelotrichaceae bacterium 21_3	4,873
12	Human / Erysipelotrichaceae bacterium 21_3	4,541
13	Human / Paraprevotella xylaniphila YIT 11841	4,366
14	Bacteroides sp. D22 / Bacteroides xylanisolvens SD CC 1b / Bacteroides xylanisolvens SD CC 2a	4,053
15	Human / 51873	4,031
16	Hafnia alvei ATCC 51873	3,947
17	Human / Klebsiella pneumoniae subsp. pneumoniae WGLW3	3,610
18	Human / Bacteroides sp. D22 / Bacteroides xylanisolvens SD CC 1b / Bacteroides xylanisolvens SD CC 2a	3,538
19	Coprobacillus sp. 8_2_54BFAA	3,442
20	Human / Faecalibacterium prausnitzii M21/2	3,414

Supplemental Figure 12. Magnification of detail in Figure 8 (Main Text).

The inset shows the upper left corner of Figure 8 around Box 3, which contains motifs unique to *Brucella melitensis*. The black bordered square is expanded to the underlying image to show individual small rectangles, each of which comprises a group of motifs which have a unique sharing relationship among the Pathogen set. Overall Figure 8 comprises over 1.5 million such rectangles.

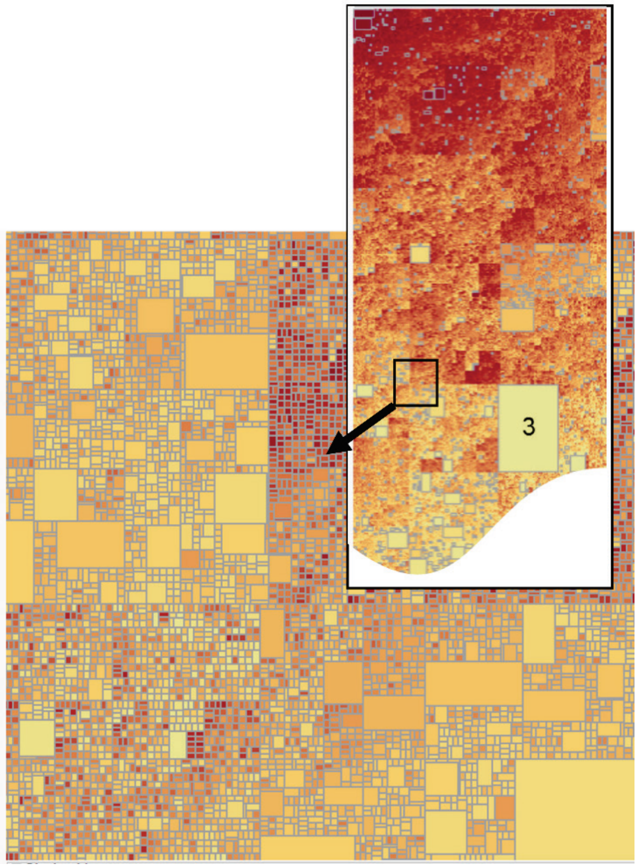

Supplement: Supplementary file 1 [file Data_Sheet_1.PDF]
